# Engineering *Saccharomyces Cerevisiae* With Novel Functional Xylose Isomerases From Rumen Microbiota for Enhanced Biofuel Production

**DOI:** 10.1002/biot.70050

**Published:** 2025-06-09

**Authors:** Beatriz de Oliveira Vargas, Marcelo Falsarella Carazzolle, Juliana Pimentel Galhardo, Juliana José, Brenda Cristina de Souza, Jéssica Batista de Lima Correia, Jade Ribeiro dos Santos, Gonçalo Amarante Guimarães Pereira, Fellipe da Silveira Bezerra de de Mello

**Affiliations:** ^1^ Departamento de Genética Evolução Microbiologia e Imunologia UNICAMP Campinas São Paulo Brazil

**Keywords:** *Saccharomyces cerevisiae*, second‐generation ethanol, xylose isomerase

## Abstract

Xylose metabolism in *Saccharomyces cerevisiae* remains a significant bottleneck due to the difficulty in identifying functional and efficient xylose isomerases (XI). In the present study, publicly available metagenomic and metatranscriptomic datasets of rumen microbiota from different herbivorous mammals were used to prospect novel XIs sequences. Seven putative XIs from moose, camel, cow, and sheep were cloned into a strain modified for xylose metabolism. Out of those, five XIs demonstrated activity and efficiently converted xylose into xylulose, resulting in ethanol as the final product. A XI from camel rumen microbiota exhibited a K_M_ of 16.25 mM, indicating high substrate affinity. The strains expressing enzymes XI11 and XI12, obtained from sheep rumen microbiota, were able to deplete 40 g/L of xylose within 72 and 96 h, achieving theoretical ethanol yields of 90% and 88%, respectively. These results are comparable to those obtained with *Orpinomyces sp*. ukk1 XI, a benchmark enzyme previously reported as highly efficient in *S. cerevisiae*. This study also provides the first report on the successful expression of XIs mined from the ruminal microbiotas of sheep and camels in *S. cerevisiae*, expanding the perspectives for the optimization of fermentation processes and the production of lignocellulosic biofuels from xylose.

## Graphical Abstract and Lay Summary

This study identified novel xylose isomerases (XIs) from ruminal microbiota using omics data. Five *Bacillota* XIs were efficiently expressed in *Saccharomyces cerevisiae*, enabling effective conversion of xylose into xylulose and subsequent ethanol production. These findings underscore the potential of omics‐based approaches to enhance lignocellulosic biofuel production from xylose.

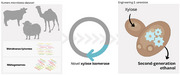

## 1 Introduction

Lignocellulosic material, an abundant and low‐cost raw feedstock, continues to be one of the main renewable sources for the production of biofuels and bioproducts [1, 2]. Its composition includes the pentose sugar xylose, which is present in high abundance in biomass [3]. While the yeast *Saccharomyces cerevisiae* has been widely applied as a platform in the industrial transformation of sugars derived from lignocellulose due to its robustness, tolerance to inhibitors, and high ethanol productivity [4, 5], it cannot naturally ferment xylose. Nevertheless, this yeast can metabolize xylulose, its isomer [6], making the expression of heterologous pathways for xylose utilization—such as xylose isomerase (XI) expression—necessary to overcome this limitation [7, 8]. Therefore, the identification of XI with high activity in *S. cerevisiae* is crucial for the viability of second‐generation biorefineries [9, 10].

In 1953, the enzyme XI, found mainly in bacteria and some fungi, was first reported to catalyze the natural conversion of xylose to xylulose [11]. However, initial attempts to functionally express XIs in *S. cerevisiae* failed [12, 13]. It was only in 1996 that an active expression of XI from the thermophilic bacterium *Thermus thermophilus* in *S. cerevisiae* was reported. However, the optimum temperature for this XI is 85°C, and its activity decreased drastically at 30°C, which made its use unfeasible in industrial fermentations [14]. The report that XIs could be functionally expressed in *S. cerevisiae* was promising, intensifying the search for more efficient variants from that point onward [15, 16].

In 2003, the first XI capable of effectively fermenting xylose at 30°C in *S. cerevisiae* was obtained from the anaerobic fungus *Piromyces sp*. E2, isolated from Indian elephant feces [17]. After extensive metabolic engineering of the host strain, the consumption of 20 g/L of xylose and an ethanol theoretical yield of 85% was achieved [18]. Subsequently, XI from the fungus *Orpinomyces sp*. ukk1, isolated from bovine rumen, and the anaerobic bacterium *Clostridium phytofermentans*, were also functionally expressed in this yeast. Modified strains revealed consumption of 15.5 and 18 g/L of xylose, with ethanol yields of approximately 76% and 84%, respectively [7, 16]. Later, in 2013, the XI from *Prevotella ruminicola*, a microorganism common in bovine rumen and intestine, demonstrated the capacity to consume 11.7 g/L of xylose with a yield of 68.6% [15].

Despite the advances, only about 20 XI variants have demonstrated significant functional activity in *S. cerevisiae* to this day, with few allowing complete and fast xylose depletion [7, 15‐17, 19, 20]. Previous studies have shown that the gastrointestinal tract of some species, especially those adapted to diets rich in lignocellulosic material, have a variety of enzymes that play essential roles in the hydrolysis of complex molecules and in the conversion of simple sugars [21, 22, 23], such as xylose, into metabolizable compounds [16, 24]. Therefore, a significant strategy for prospecting XIs has been the exploration of metagenome libraries, which enabled the identification of XIs from sources such as soil [25], bovine rumen [24], and the intestine of the wood‐feeding beetle *Odontotaenius disjunctus* [26]—all characterized by low oxygen levels and high lignocellulose degradation rates. Metagenomic and metatranscriptomic approaches have proven to be robust tools for discovering and characterizing new enzymes, in addition to allowing a comprehensive analysis of the genetic diversity present in different microbiomes [27, 28].

In this study, we propose the prospecting and characterization of novel XIs through the screening of public metadata libraries from the rumen microbiota of herbivorous mammals. For this approach, 11 datasets were used: four metatranscriptomes from cows and sheep, and seven metagenomes from goats, moose, manatees, and camels. Approximately 52 million transcripts and genes were analyzed, resulting in the selection of seven putative XIs, optimized and cloned in *S. cerevisiae*. Five of those sequences demonstrated activity and allowed the metabolization of different concentrations of xylose, without the need for adaptive evolution of the host strain. These results indicate that the new sequences represent a discovery with great potential, expanding the knowledge about xylose conversion in *S. cerevisiae*, in addition to having a significant impact on the lignocellulosic biofuels industry.

## 2 Materials and Methods

### 2.1 Sequence Prospection

For the analysis, 11 sequenced datasets from the rumen of herbivorous mammals were selected based on their large number of transcripts/genes and their respective translated proteins, present in the Integrated Microbial Genomes and Microbiomes (IMG/M) database at the Joint Genome Institute (JGI) (https://img.jgi.doe.gov/cgi‐bin/m/main.cgi). These datasets include four metatranscriptomes from cows (ID 3300009872 and 3300033463) and sheep (ID 3300033463 and 3300001486), in addition to seven metagenomes from goats (ID 3300001395 and 3300001425), moose (ID 3300011008 and 3300010998), manatee (ID 2124908003), and camels (ID 3300003523 and 3300002597). Using a set of functionally expressed XIs proteins that were prospected and published by [10], a sequence domain profile based on hidden Markov models (HMM profile) was constructed using the HMMER 3 package [29]. Then, this HMM was used to prospect the XIs proteins in the JGI datasets using hmmsearch with a threshold E‐value of 1e‐2, followed by identification of the gene and transcript sequences encoding these proteins. To eliminate sequence redundancy, the CD‐HIT program [30] was used for clustering the protein sequences with a sequence identity threshold of 0.95, and the representative sequence for each cluster shorter than 400 aa was discarded, as representing an incomplete sequence. Finally, the filtered proteins were used to select sequences with freedom to operate (patent‐free) through alignment with the NCBI patent sequence database using the Basic Local Alignment Search Tool (BLASTp) with a threshold E‐value of 1e‐5 and sequence identity ≤ 70%.

### 2.2 Phylogenetic Analysis

Phylogenetic inference was performed to assess the diversity of the XIs enzymes prospected in the present work. The initial dataset consisted of amino acid sequences of: (i) XIs protein present in this work; (ii) functional XIs sequences phenotyped by [19], and (iii) XIs sequences explored by [26]. Protein sequences that failed the composition test implemented on IQ‐tree v2.1.2 [31] were discarded, totaling 45 sequences in the final dataset (Table S7). Initially, protein alignment was obtained with MAFFT v7.271 [32], using up to 1000 iterations and the L‐INS‐i algorithm, which allows long gaps on sequences with conserved domains [33]. Finally, phylogeny was inferred using maximum likelihood methods implemented in IQ‐tree v2.1.2 [31] using 1000 bootstraps for branch support and the best‐fit model LG+I+G4, chosen according to BIC by IQ‐tree tests. XIs functionality and taxonomic annotation are also depicted along with the phylogenetic tree. The enzyme's functionality on *S. cerevisiae* is treated as a binary trait, and the information was obtained from [19, 26], and experimental data from the present work. The GTDB database (https://gtdb.ecogenomic.org/) was used to access taxonomic information from sequences not yet identified.

### 2.3 Strains, Media, and Plasmids

For the characterization of the novel XIs, *S. cerevisiae* strains GGY018 and BVY271 were used. Growth and maintenance of yeast strains were performed in YP medium (10 g/L yeast extract and 20 g/L peptone) or YNB medium (6.7 g/L yeast nitrogen base supplemented with amino acids), both containing 20 g/L of glucose, at 30°C and 200 rpm. No uracil was added to the YNB medium (YNB URA‐) for XIs vectors selection. Hygromycin B (300 µg/mL) or geneticin G418 (200 µg/mL) was added to the YPD medium for the selection of transformant strains carrying the *hphMX6* or *KanMX* resistance markers, respectively. The new XIs sequences and the positive control (*Orpinomyces sp*. ukk1 *xylA*) were codon‐optimized for expression in *S. cerevisiae*, synthesized, and cloned into the XbaI‐XhoI site of the pRS426_GPD vector [34]. The plasmids were propagated in *Escherichia coli* DH5α grown in LB medium (10 g/L tryptone, 10 g/L NaCl, and 5 g/L yeast extract) supplemented with 100 µg/mL ampicillin (LB‐amp), either as liquid or solid medium on plates containing 20 g/L agar. Transformation of DH10β *E. coli* cells was performed using the electroporation protocol [35]. Extraction of bacterial plasmids was performed using a standard miniprep protocol [36]. *E. coli* cultures in LB medium were grown at 37°C and 250 rpm in shaking incubators for liquid cultures or in static incubators for solid cultures. All yeast strains and plasmids used in this study are listed in Table S1.

### 2.4 Genetic Engineering

Strain GGY018 is a haploid obtained from a hybrid of the bioethanol industrial strains PE‐2 and SA‐1 [37], engineered for xylose fermentation. The four genes of the non‐oxidative pentose phosphate pathway (PPP) were amplified from the genome of *S. cerevisiae* LVY27 [38], modified for xylose consumption, and inserted by homologous recombination into CEN12 and CEN13, using pAG32 [39] for the selection of transformants. *GRE3* was disrupted by insertion of a hygromycin B resistance cassette, amplified from plasmid pAG32 by polymerase chain reaction (PCR) with primers containing ends with homology outside the gene's open reading frame (ORF). *URA3* knockout was performed using a CRISPR‐Cas9 system as described by [40]. For that, a 90‐bp double‐strand oligo with homology to the target site, containing a stop codon that replaces the PAM, was transformed into the strain combined with a Cas9‐sgRNA vector (pGS). Transformants carrying the pGS plasmid were selected in geneticin. Strain BVY271 was constructed from LVY27. The *Orpinomyces sp*. ukk1 *xylA* present in CEN5 was inactivated by knock‐in of *hphMX6* cassette, as previously described. Deletion of *URA3* followed the same approach used in strain GGY018. All modifications were confirmed by PCR or Sanger sequencing. Yeast transformations were performed using the LiAc/SS carrier DNA/PEG method [41]. DNA extractions were performed by the rapid (LiOAc)‐SDS/EtOH protocol [42]. All PCR reactions were performed with Phusion High Fidelity DNA Polymerase, following the manufacturer's instructions (ThermoFisher). Primers used are listed in Table S2.

### 2.5 Growth Characterization in Microplates

Semi‐anaerobic microculture screening was performed at 30°C using a microplate reader (Spectramax 384 Plus, Molecular Devices). Cells were pre‐cultured overnight in selective YNB URA—and subsequently washed with sterile water. An initial optical density (OD_600_) of 0.1 was used for all microplate assays. The experiment was performed in 96‐well flat‐bottom microtiter plates sealed with MicroAmp translucent film, containing 135 µL of culture medium and 15 µL of inoculum at OD_600_ 1.0, at least in quadruplicates. OD_600_ readings were taken at 180 min intervals for a total of 216 h. Analysis of growth kinetic parameters was performed using OCHT software [43]. For graphic construction, the Python packages pandas, matplotlib, and NumPy were used [44, 45, 46, 47]. Control cultivations were performed in either YP or YNB URA—supplemented with 20 g/L glucose. For treatments, 50 g/L xylose was added to the minimal (YNBX) and rich (YPX) media, without glucose. In tests with metals, iron sulfate (100 µM), magnesium sulfate (500 µM), and manganese sulfate (100 µM) were added to the YNBX medium [38].

### 2.6 Enzymatic Activity

Yeast cells expressing the XI‐containing plasmids were grown in YNB URA‐ medium supplemented with glucose overnight for pre‐inoculum. The cultures were diluted in 50 mL of the same medium to an OD_600_ of 0.3 and harvested in the mid‐log phase, centrifuged, washed with sterile Milli‐Q water, and resuspended in Y‐PER (Thermo Fisher Scientific) according to the manufacturer's instructions. The cells were incubated at room temperature for 20 min at 200 rpm. Next, the samples were centrifuged at 2000 g for 5 min. The supernatant was carefully collected and kept on ice to preserve its integrity. Protein concentration was measured in NanoDrop at 340 nm, and enzymatic assays were performed immediately after sample preparation. The enzymatic activity of XIs was performed as described elsewhere by [26], adapted for microplate, using 100 mM Tris‐HCl buffer (pH 7.5), 10 mM MgCl_2_, 0.15 mM NADH, 2 U sorbitol dehydrogenase (Sigma‐Aldrich), and 1 mg/mL of cell lysate. The reactions were initiated by the addition of D‐xylose to a final concentration of 600 mM. The activity was determined by monitoring the oxidation of NADH at 340 nm for 30 min at 30°C using a microplate reader. The specific activity of the enzymes is expressed in U·(mg of protein)⁻¹, where 1 U corresponds to the conversion of 1 µM of substrate per minute, under the assay conditions. All enzymatic assays were performed in triplicate. The kinetic parameters were obtained by interpolating the experimental data in the Michaelis–Menten curve, using the least squares fitting method.

### 2.7 Fermentation

Semi‐anaerobic batch fermentation experiments were performed at 30°C and 150 rpm in Erlenmeyer flasks (250 mL) sealed with rubber stoppers containing 100 mL of YP with 5 g/L glucose and 40 g/L adapted xylose [38]. Pre‐inoculum cells cultivated in YNB URA‐ were washed with sterile water and inoculated at an OD_600_ of 1.0. Experiments were performed in triplicate, and samples were collected at 0, 4, 8, and subsequently every 24 h, totaling 216 h, for cell density and metabolite analysis. The xylose and ethanol concentrations in the samples were determined by high‐performance liquid chromatography (HPLC) using a Bio‐Rad HPX‐87H column, using 5 mM sulfuric acid as the mobile phase and a flow rate of 0.6 mL/min. The components were detected using a refractive index detector, with a controlled temperature of 35°C.

### 2.8 Statistical Analysis and Fermentation Parameters Calculation

Fermentation parameters were calculated as follows: xylose conversion (%) was determined by dividing the amount of xylose consumed (g/L) by the initial concentration (g/L). Ethanol yield (g/g) was calculated by the ratio between the amount of ethanol produced (g/L) and the total amount of sugar consumed (g/L), including both glucose and xylose. The values ​​obtained by HPLC were considered in the calculations. The data from the experiments performed were analyzed and considered statistically different with a 95% confidence interval, using the Analysis of Variance (ANOVA) test (*p* < 0.05) and Tukey's test when data residuals were normally distributed. The results presented are the mean values ​​of the replicates of the tests with standard error values. All data are presented as mean ± standard deviation.

## 3 Results and Discussion

### 3.1 Prospection and Taxonomical Identification of XIs Gene Candidates

**FIGURE 1:**
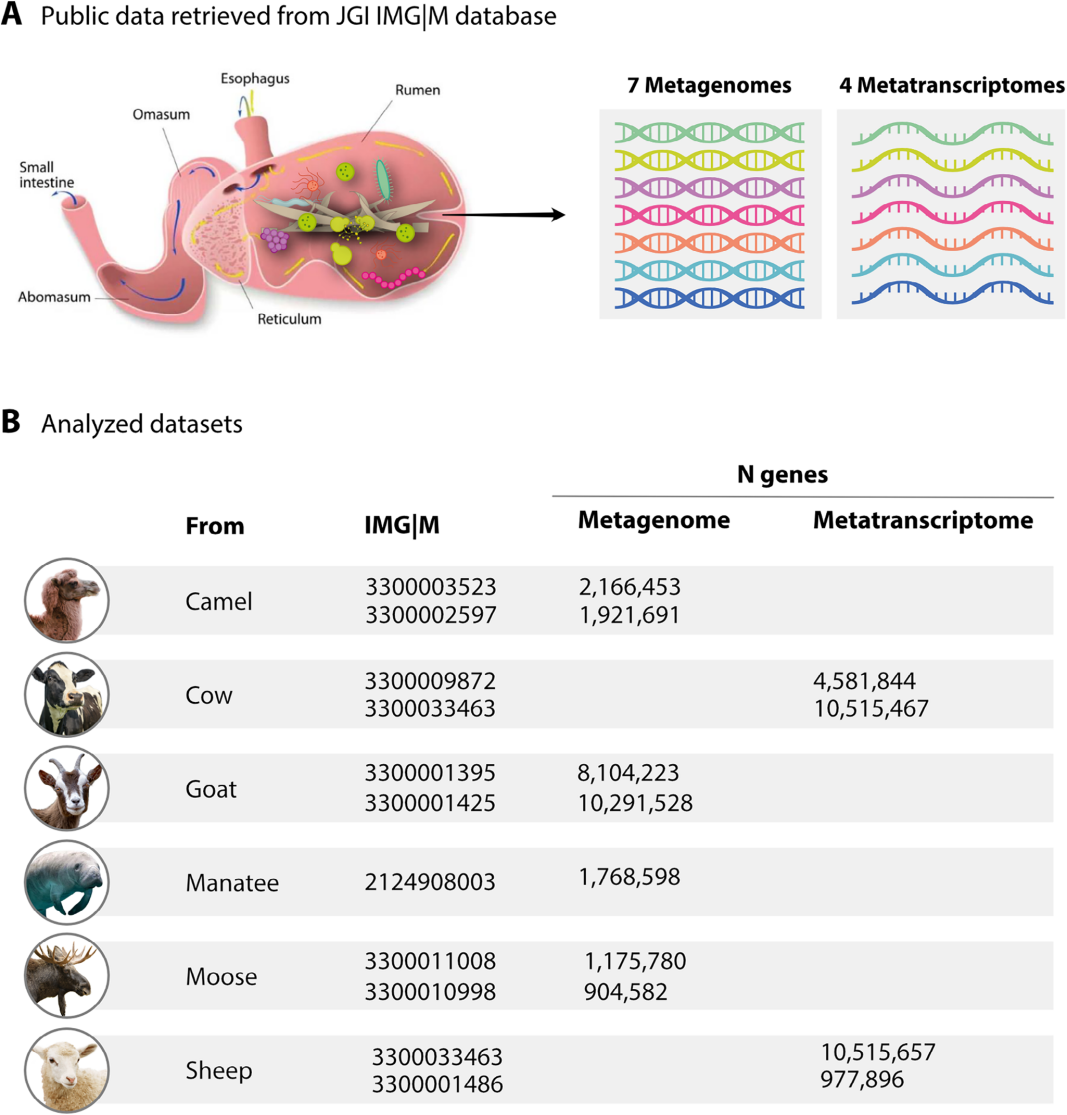
Herbivorous mammals’ rumen microbiota datasets from public metagenomic and metatranscriptomics studies used for xylose isomerase gene prospecting. (a) Seven metagenomic and four metatranscriptomic datasets of ruminal microorganisms were selected from the Integrated Microbial Genomes and Microbiomes (IMG/M) database of the Joint Genome Institute (JGI) for XIs prospection; b) Datasets analyzed according to the animals to which they belong, the respective identification of the datasets and number of genes/transcripts in each study.

There is still no standard protocol for identifying functional XIs in *S. cerevisiae*. Endeavours usually rely on approaches ranging from random mutagenesis through degenerate PCR [15, 16, 17] to the use of omics techniques for the prospection of new sequences [26, 48]. In this scenario, our efforts were directed at screening public metagenomic and metatranscriptomic datasets of the rumen microbiota of herbivorous mammals deposited in the IMG/M of the JGI portal, in order to identify novel XIs (Figure 1A). For this analysis, 11 datasets, including metagenomes and metatranscriptomes, were analyzed: four cow and sheep metatranscriptomes, and seven goats, moose, manatee and camel metagenomes (Figure 1B), resulting in a total of 26,332,855 predicted genes and 26,590,864 transcripts, and their respective translated proteins, for the metagenomic and metatranscriptomics data, respectively. The prospection of XIs genes was performed based on HMM search, resulting in 2.846 candidates, which were reduced to 151 sequences after the clustering step with 95% identity and a minimum length of 400 aa. Finally, after filtering by patentability criteria, the final dataset comprised seven putative new XIs genes. The newly selected sequences are provided in Table S3.

The selected novel XIs were named XI09, XI10, XI11, XI12, XI13, XI14, and XI15. Among these, five (XI09, XI10, XI11, XI12, and XI13) were obtained from metatranscriptomes of the rumen microbiota of sheep and cow, with XI10, XI11, XI12, and XI13 obtained from the analysis of the sheep dataset (3300033463 and 3300001486), and only XI09 from the cow library (3300009872 and 3300033463). XI14 and XI15 were prospected in metagenomic data of the rumen microbiota of moose (3300011008 and 3300010998) and camel (3300003523 and 3300002597), respectively.

**FIGURE 2:**
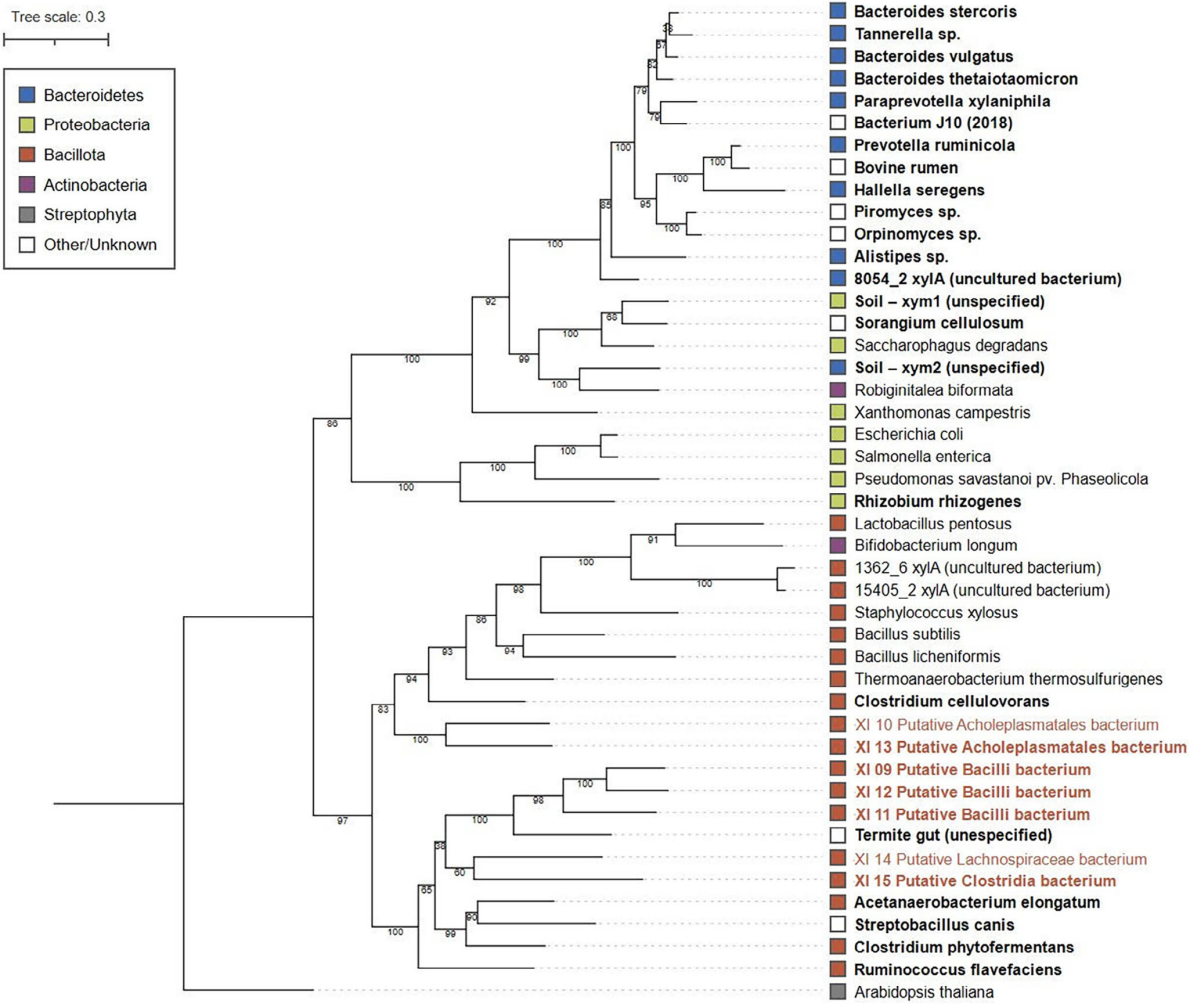
Phylogenetic inference of Xylose Isomerase proteins and functionality mapping. The tree was reconstructed using the Maximum Likelihood method, branch support was determined by posterior probability, and branches’ lengths corresponded to the proportion of substitutions. Color code represents taxonomic annotation, and bold names correspond to XI functional in *S. cerevisiae*, as referred to in the literature and further in this study. XI's 09 to 15 are the ones prospected and phenotyped in the present work.

The putative taxonomy identification of each XI protein sequence was assessed by BLASTp search. XI09, XI11, and XI12 present the *Bacilli* class (98.86%, 97.96%, and 87.93% identity). XI10 and XI13 harbor on *Acholeplasmataceae* order (76.15% and 84.90% identity); and XI14 and XI15 are native to the *Lachnospiraceae* family and the *Clostridia* genus, respectively (99.77% and 84.30% identity). To determine whether the sequences prospected in this work are closely related to previously described xylose isomerases, the phylogenetic inference was performed (Figure 2). As expected by the similarity analysis, XI09, XI10, XI11, XI12, XI13, XI14, and XI15 are from phylum *Bacillota* (previously known as *Firmicutes*) and cluster along with other XIs prospected from species of this taxon. Even though an enrichment of functional XIs has been related to enzymes from *Bacteroidetes* rather than other taxa [26], our pipeline of prospection through metadata from herbivorous rumen brought seven XIs candidates from different taxa of *Bacillota* phylum.

Despite substantial efforts, only about 20 XIs have demonstrated activity on *S. cerevisiae* (Table S7). Out of those, the most efficient are the ones prospected from fungi (*Piromyces* sp., and *Orpinomyces* sp.), and *Clostridium phytofermentans*, a representative from the *Bacillota* phylum—all used in industrial processes for lignocellulosic ethanol production [38, 49]. Although higher enzymatic activity is observed on XIs isolated from *Bacteroidetes* or fungi, all functional XIs prospected in the present work (XI09, XI11, XI12, XI13, and XI15) are from the phylum *Bacillota*.

### 3.2 Novel Set of Functional XIs in *S. cerevisiae* Is Uncovered

To assess the prospected XIs functionality in *S. cerevisiae*, a preliminary growth assay was performed in microplates to identify the best candidates for further analysis. All novel *xylA* genes were codon‐optimized and cloned in plasmid pRS426_GPD [34]. The XI‐expressing vectors were transformed into the industrial strain GGY018, previously constructed for xylose consumption. The host strain was selected for harboring overexpression of the four genes of the PPP (*TAL1*, *TKL1, RKI1*, and *RPE1*), *GRE3*, and *URA3* knockout, common modifications for xylose fermentation in *S. cerevisiae* [50]. The growth of each XI's candidate was compared to *Orpinomyces sp*. ukk1 XI (OrpXI) and the empty pRS426 (EV), without XI expression. Cultures were tested in both complex (YPX) and minimal (YNBX) media containing 50 g/L of xylose in semi‐anaerobiosis in 96‐well plates. The results are presented in Figure S1 and Table S4.

The negative control culture (EV) did not show an expressive increase in cell density in either medium used, reaching an OD_600_ of 0.14 ± 0.06 in YNBX and 0.30 ± 0.01 in YPX, respectively, after 216 h of growth. This result reinforces that, even with the overexpression of genes related to the metabolic flux of xylose, XI's presence is essential for yeast's significant growth. The strains containing the enzymes XI10 (sheep rumen) and XI14 (moose rumen) showed statistically similar growth to the strain expressing EV in both culture conditions, suggesting that these enzymes are not functional in *S. cerevisiae*. On the other hand, the strain expressing OrpXI, as well as those containing enzymes XI09, XI11, XI12, XI13, and XI15, were able to grow on xylose as the sole carbon source, indicating that they are functional in *S. cerevisiae*. However, growth rates were slow, with final OD_600_ ranging from 0.29 to 0.52 in YNBX and 0.52 to 0.83 in YPX.

Within the *Bacillota* phylum, to which all the microorganisms of the selected XIs belong, a variation in the functionality of this enzyme has been observed (Figure 2), with some strains presenting functional enzymes and others not when expressed in *S. cerevisiae* [7, 12, 19, 51]. XIs inefficient expression in *S. cerevisiae* has been previously associated with environmental factors (pH, temperature, and availability of metal ions), differences in protein synthesis, and enzyme folding [13, 52]. Inappropriate or redundant N‐terminal sequences in XIs have also been linked to inactivity in *S. cerevisiae* [19, 53].

When strains expressing XI09, XI11, XI12, XI13, and XI15 were individually compared between treatments, a substantial increase in the final OD_600_ of 79.31%, 64.29%, 59.92%, 41.03%, and 37.21%, respectively, was observed between growth in minimal and complex medium. These results suggest that increased nutritional and cofactor availability can significantly improve host strain performance and the expression of new enzymes. These findings corroborate previous studies that indicated that medium composition can have a significant impact on enzyme efficiency and yeast performance [54]. In this sense, for subsequent investigations, only the five sequences (XI09, XI11, XI12, XI13, and XI15) that presented significantly higher growth than the negative control in both tested conditions were selected, according to the statistical analysis performed by the ANOVA test (*p* < 0.05).

### 3.3 Five Novel XI Allows Substantial Growth in Xylose

**FIGURE 3:**
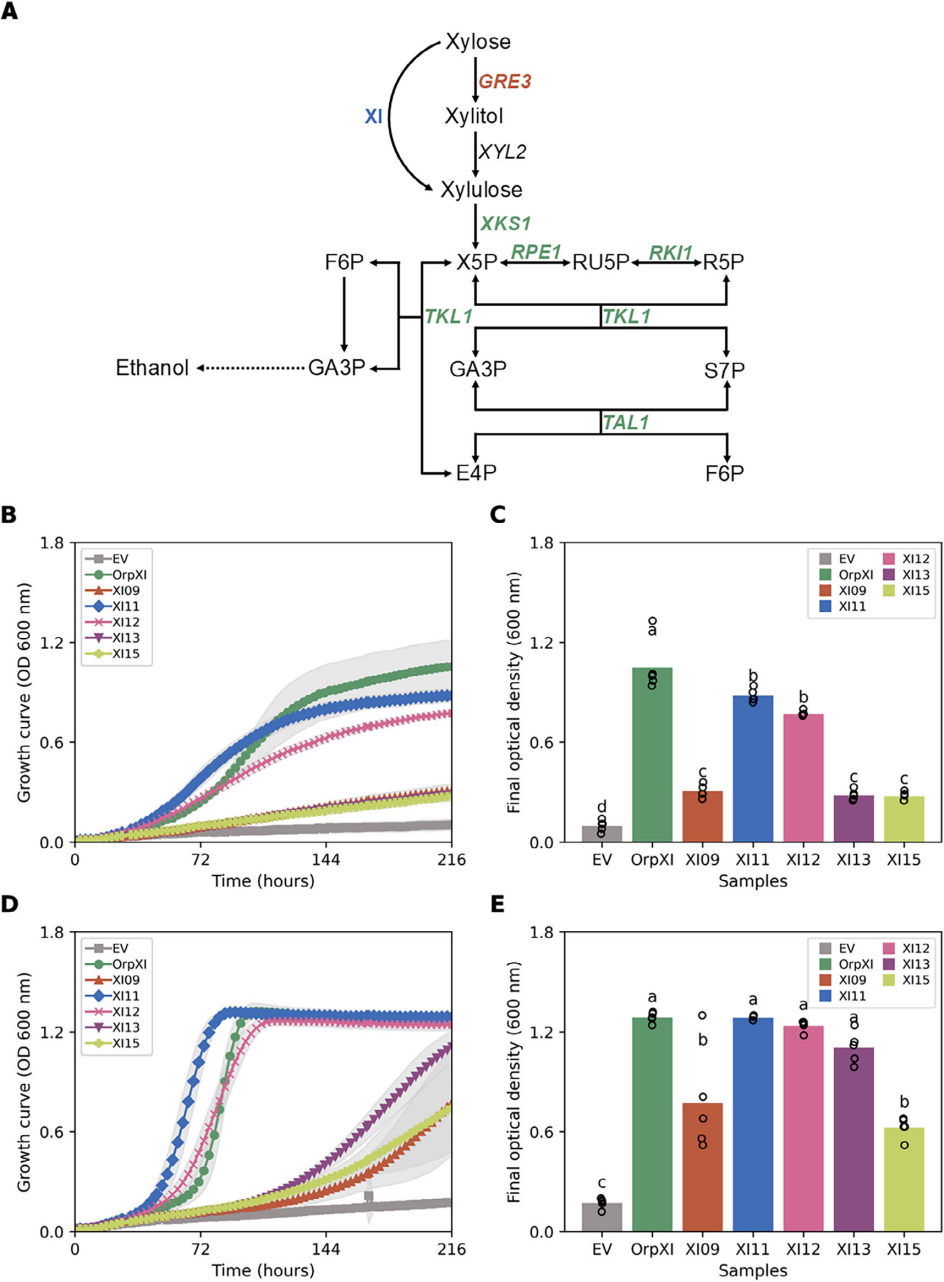
*Saccharomyces cerevisiae* BVY271 expressing various xylose isomerases identified through metagenomic and metatranscriptomic analysis of the rumen microbiota of herbivorous mammals. (A) Metabolic engineering of the BVY271 strain for xylose metabolism via the isomerase pathway; in green, genes that are overexpressed; orange, deleted genes; and in blue where the XIs are located in the xylose consumption pathway; XYL2 = xylose dehydrogenase; X5P = xylulose 5‐phosphate; RU5P = ribulose 5‐phosphate; R5P = ribose 5‐phosphate; GA3P = glyceraldehyde 3‐phosphate; S7P = sedoheptulose 7‐phosphate; E4P = erythrose 4‐phosphate and F6P = Fructose 6‐phosphate. (B) Growth curves in minimal medium containing 50 g/L xylose (YNBX) over 216 h. (C) Final optical density (OD_600_) after 216 hours of growth in YNBX. (D) Growth curves in rich medium containing 50 g/L xylose (YPX) over 216 hours. (E) Final OD_600_ after 216 h of growth in YPX. In panels (B), (C), (D) and (E), legends represent the names of the tested samples, with the negative control shown as the empty vector (EV) and the positive control as *Orpinomyces* sp. *xylA* (OrpXI). In (B) and (D), dark lines represent the sample data points, and shading represents the standard deviation in relation to the mean of the samples. In panels (C) and (E), different letters denote statistically significant (*p* < 0.05) differences between the samples for each evaluated parameter, as determined by ANOVA, and circles represent the data points. The experiment was performed in quintuplicate.

For subsequent assays, the selected sequences XI09, XI11, XI12, XI13, and XI15, together with the controls, were expressed and re‐evaluated under the same conditions as the previous experiment—cell growth in microplates under minimal and complex media with 5% xylose—but now in strain BVY271. This host strain, derived from LVY27 [38], has an industrial background and presents the same genetic modifications present in GGY018, together with *XKS1* overexpression (Figure 3A). This gene encodes xylulokinase, an enzyme responsible for the phosphorylation of xylose into xylulose‐5‐phosphate, accelerating the metabolic flux of xylose consumption in yeast [55]. The *Orpinomyces sp*. ukk1 *xylA* gene present in LVY27 was knocked out, as well as *URA3*, for auxotrophy. When only glucose is available in the medium, all transformed BVY271 strains performed similarly (Figure S2 and Table S5).

In microculture assays with strain BVY271 expressing enzymes XI09, XI11, XI12, XI13, and XI15 in YNBX (Figure 3B,C and Table S5), final OD_600_ values ​​obtained after 216 h of growth ranged between 0.28 and 0.88, significantly higher than the EV control (0.10 ± 0.03), but still lower than the strain expressing OrpXI (1.05 ± 0.14). In YPX (Figure 3D,E and Table S5), OD_600_ values for cells expressing the novel xylose isomerases varied from 0.75 to 1.29, well above 0.17 ± 0.02 obtained by the EV‐expressing cells. In the complex medium, strain containing OrpXI presented a final OD_600_ of 1.29 ± 0.02. Notably, the sequences of XI11, XI12, and XI13 allowed growth with a final OD_600_ statistically similar to that of OrpXI in the same period, according to the ANOVA test (*p* < 0.05). In contrast, the strains containing XI09 and XI15 presented final OD_600_ 1.65‐fold and 1.73‐fold lower than OrpXI, respectively.

In addition to improving biomass titer, the YPX medium also favored the adaptation time of cells, since BVY271 expressing XI11 and XI12 presented an OD_600_ of 1.10 ± 0.1 and 0.47 ± 0.08 after 72 h of growth, respectively, both higher than that of OrpXI (0.30 ± 0.08) in the same period. These results suggest that XI11 and XI12 allow xylose metabolization 3.67‐fold and 1.57‐fold faster, respectively, than OrpXI. On the other hand, in 72 h, the cells expressing XI09, XI13, and XI15 presented OD_600_ of 0.11 ± 0.01, 0.11 ± 0.01, and 0.12 ± 0.02, respectively, indicating a slower adaptation—slightly higher than EV (0.08 ± 0.01).

Overall, strain BVY271 outperformed GGY018 regarding biomass production. Such phenotype variation amongst strains expressing the same heterologous enzymes may be attributed to modifications in the intracellular and post‐translational differences, effects that are directly related to the intrinsic characteristics of the strain used [19]. The trait variation between strains may also be associated with *XKS1* overexpression, present in BVY271, underscoring its importance in xylose metabolism in *S. cerevisiae*. Finally, the results suggest that a robust host strain, with metabolic pathways optimized for xylose metabolism, associated with a higher availability of nutrients and cofactors, can significantly improve the performance of the tested enzymes. This observation highlights the importance of selecting suitable strains, metabolic engineering, and adjusting culture conditions when evaluating potentially functional enzymes.

### 3.4 Addition of Iron in Medium Improves XIs Efficiency

**FIGURE 4:**
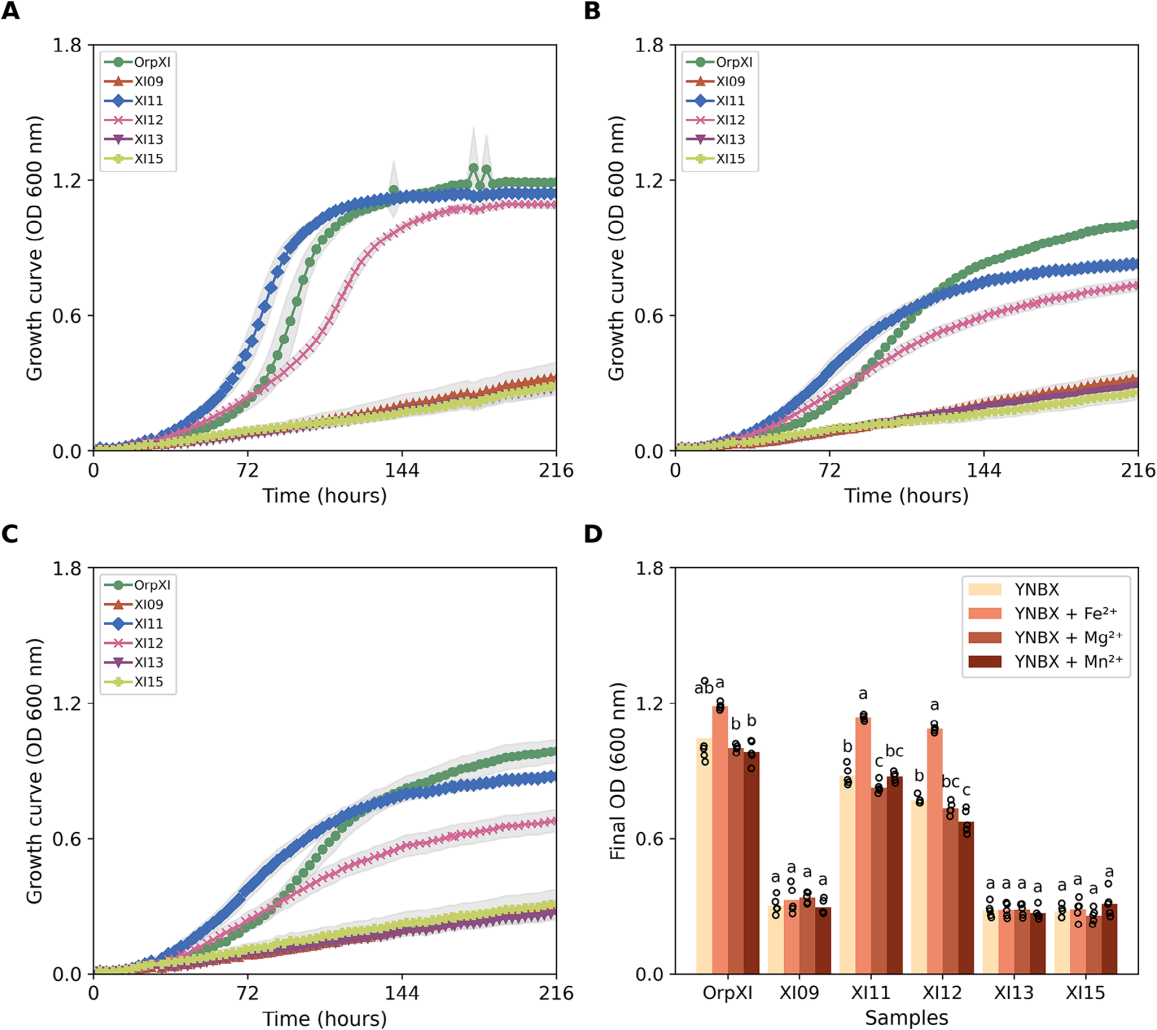
*Saccharomyces cerevisiae* BVY271 expressing different xylose isomerases in microculture with minimal medium containing 50 g/L xylose (YNBX) and supplementation with divalent metal ions. Growth curves of the strains over 216 h with the addition of Fe^2^⁺ (A), Mg^2^⁺ (B), and Mn^2^⁺ (C). The captions in the figures indicate the names of the tested samples, with the negative control represented by the empty vector (EV) and the positive control as *Orpinomyces sp. xylA* (OrpXI). Dark lines represent the sample data points and shading represents the standard deviation in relation to the mean of the samples in panels (A), (B), and (C). In panel (D), the circles indicate the data points and different letters indicate statistically significant values ​​(*p* < 0.05) among the samples for each parameter evaluated, as determined by ANOVA. The experiment was performed in quintuplicate.

XIs are tetrameric enzymes and require divalent metals to promote the enzymatic catalysis of xylose into xylulose. These divalent metals perform two functions: the first metal is related to the structure of the enzyme, facilitating the correct coupling of the substrate, while the second is a catalytic cofactor, essential for the isomerization process [56]. Although XIs present high conservation in the region of the active sites, the affinity for metals is individual for each enzyme [57, 58]. Previous studies have shown that the addition of divalent metals in media exerts a significant effect on the enzymatic catalysis of isomerases [57, 59]. In this context, microculture assays were performed to investigate whether supplementation with metal ions Fe^2^⁺, Mg^2^⁺, and Mn^2^⁺, the most frequently required metal cofactors for XIs activity, could improve the growth of strains expressing the novel enzymes. The culture was monitored under the same conditions as in the previous experiments, using the minimal medium YNBX (50 g/L xylose) supplemented with Fe^2^⁺ (100 mM), Mg^2^⁺ (500 mM), or Mn^2^⁺ (100 mM), as described by Dos Santos et al., [38]. The final OD_600_ after 216 h of cultivation was used as a parameter to evaluate the effect of metal ions on the efficiency of XIs. Results are presented in Figure 4 and Table S6.

The positive control strain expressing OrpXI, as well as those with isomerases XI11 and XI12, reached final OD_600_ of 1.19 ± 0.01, 1.14 ± 0.01, and 1.09 ± 0.01, respectively, with Fe^2^⁺ supplementation, which represented a significant increase of 1.13‐fold, 1.29‐fold and 1.41‐fold increases in relation to the non‐supplemented minimal medium. Furthermore, iron supplementation reduced the adaptation time of the yeast expressing XI11 by 1.6‐fold, allowing it to reach OD_600_ of 0.93 ± 0.03 in 96 h, while in the non‐supplemented medium, an OD_600_ of 0.58 ± 0.04 was observed for the same time period. These results corroborate the findings of Dos Santos et al. [38], who observed that the addition of Fe^2^⁺ increased the xylose consumption rate in LVY27, the parental strain of BVY271, also expressing an *Orpinomyces sp*. ukk1 *xylA*. Although residual xylose concentrations were not quantified, the observed increase in biomass likely reflects more efficient substrate utilization, indirectly supporting the stimulatory role of Fe^2^⁺ ions in the activity of XI11 and XI12. Thus, Fe^2^⁺ supplementation in fermentation processes may be beneficial for the performance of these enzymes and, consequently, for ethanol production. On the other hand, no statistically significant difference was observed in biomass production ​​between treatments for the BVY271 strains expressing XI09, XI13, and XI15. Therefore, additional tests are needed to better understand the effect of metal supplementation on the newly identified XIs, expanding the experiments to combine more metals already tested or exploring other divalent metal ions, such as Co^2^⁺, Zn^2^⁺, and Ca^2^⁺ [57, 60].

### 3.5 Enzymatic Characterization of Novel XI

**FIGURE 5:**
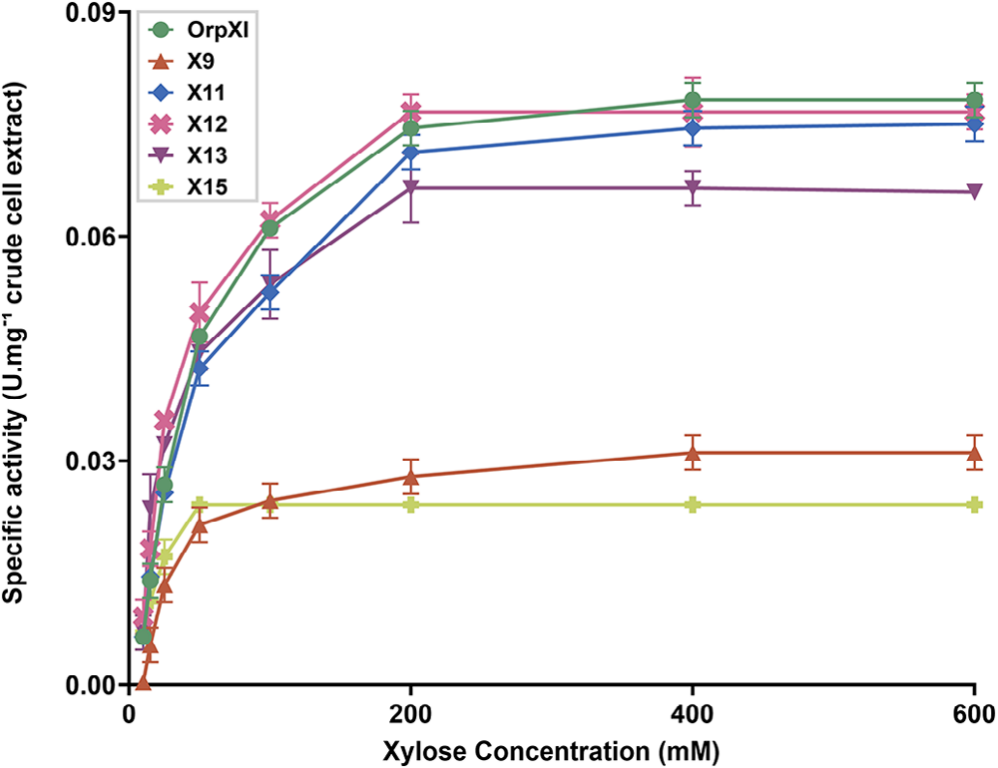
Specific enzymatic activity of different xylose isomerases in crude cell extracts. Kinetic parameters were determined from a Michaelis–Menten plot fitted to the experimental data (*R* squared > 0.95). Strains were grown in minimal medium (YNB) containing 20 g/L glucose until mid‐log phase. Crude cell extracts were prepared and used immediately in the activity assays. The data points represent an average of three replicates. Bars represent standard deviation between samples. The legend indicates the names of the tested samples, with the negative control represented by the empty vector (EV) and the positive control as *Orpinomyces sp*. *xylA* (OrpXI). The experiment was performed in triplicate.

Kinetic properties of the new enzymes (XI09, XI11, XI12, XI13, and XI15) and the control OrpXI were determined using crude extracts obtained from the cell lysate of recombinant strains, as previously described and adapted from [26]. A Michaelis–Menten curve was fitted to the experimental values obtained from the enzymes analyzed (Figure 5). Detailed information on the kinetic properties of the novel XIs and other reference enzymes described in the literature is found in Table 1. The extracts containing the enzymes XI13, XI11, and XI12 converted xylose at a maximum rate (V_max_) of 0.0732 U·mg^−1^, 0.0861 U·mg^−1^, and 0.0864 U·mg^−1^, respectively, values ​​close to those obtained in the positive control OrpXI (0.0901 U·mg⁻¹). These values ​​indicate that, in general, the enzymes have a moderate capacity for xylose conversion. In contrast, the V_max_ of enzymes XI09 (0.0349 U·mg^−1^) and XI15 (0.0264 U·mg^−1^) were 2.58‐fold and 3.41‐fold lower than the positive control, respectively.

**TABLE 1:** Kinetic properties of xylose isomerases heterologously expressed in *Saccharomyces cerevisiae* and characterized in crude cell extracts.

Source of XI	K_M_ (mM)	V_max_ (U·mg⁻¹)	References
***Thermus thermophilus***	NA	1.0^a^	[14]
***Piromyces sp*. E2**	20	1.1	[17]
***Piromyces sp*. E2** ^b^	49.85	0.0538	[7]
***Piromyces sp*. E2**	51	0.25	[15]
***Piromyces sp*. E2** ^b^	14.90	0.0103	[61]
***Piromyces sp*. E2** ^b^	30.76	0.0112	[26]
***Orpinomyces sp*. ukk1**	NA	1.90^c^	[16]
***Clostridium phytofermentans*** ^b^	66.01	0.0344	[7]
**Soil *xym1* (unspecified)**	NA	0.33	[25]
**Soil *xym2* (unspecified)**	NA	0.20	[25]
***Bacteroides stercoris***	54.03	NA	[62]
***Ruminococcus flavefaciens*** ^b^	117.1	0.29	[53]
***Prevotella ruminicola*** ^b^	40	0.28	[15]
***Bacteroides vulgatus***	NA	∼ 1.25	[20]
**Bovine rumen**	16.8	1.31	[24]
***Sorangium cellulosum***	17.2	0.35	[24]
**Termite gut (unspecified)** ^b^	10.52	0.0074	[61]
**8054_2 *xylA* (uncultured bacterium)** ^b^	19.27	0.0296	[26]
***Bacterium J10 (2018)***	NA	0.48^c^	[19]
***Hallella seregens***	NA	0.59^c^	[19]
***Acetanaerobacterium elongatum***	NA	0.37^c^	[19]
***Streptobacillus canis***	NA	0.37^c^	[19]
***Orpinomyces sp*. ukk1** ^b^	55.87	0.0901	This study
**XI09 Putative *Bacilli bacterium*** ^b^	48	0.0349	This study
**XI11 Putative *Bacilli bacterium*** ^b^	59.97	0.0861	This study
**XI12 Putative *Bacilli bacterium*** ^b^	41.9	0.0864	This study
**XI13 Putative *Acholeplasmatales bacterium*** ^b^	35.71	0.0732	This study
**XI15 Putative *Clostridia bacterium*** ^b^	16.25	0.0264	This study

Abbreviation: NA, not available.

^a^ Determined by measuring the conversion of fructose to glucose.

^b^ Codon‐optimized gene.

^c^ Determination of xylose isomerization to xylulose using High‐performance liquid chromatography.

The V_max_ value ​​obtained by OrpXI in this assay is lower than that observed in the study in which *Orpinomyces sp*. XI was identified (1.90 U·mg^−1^) [16]. The differences in the results obtained can be attributed to the distinct methods and experimental conditions adopted in each analysis. In the present study, the enzymatic assay was performed at 30°C, monitoring the reduction of NADH by spectrophotometry, using different concentrations of xylose. In contrast, the study by Madhavan et al. was conducted at 37°C, with 50 mM xylose, and the quantification of xylulose was done directly by HPLC. These variations in the methods and experimental conditions may result in differences in the precision and values ​​obtained in the analyses. Also, in the enzymatic assays of XIs, Mg^2^⁺ is generally used as a cofactor. However, the new XIs might have affinity, and consequently greater activity, in the presence of other divalent metals, such as Fe^2^⁺, as previously observed, Mn^2^⁺, and Co^2^⁺. Nevertheless, under the same conditions, here we uncover three novel XIs with V_max_ comparable to the benchmark *Orpinomyces sp. xylA*.

In comparison with other functional XIs from different sources, such as those obtained from *C. phytofermentans* (0.0344 U·mg^−1^) [7], Passalid beetle gut (8054_2) (0.0296 U·mg^−1^) [26], and *Piromyces sp*. E2 (from 0.0103 to 1.1 U·mg^−1^) [7, 15, 17, 26], the values ​​found for XIs in the present study are slightly higher.

Regarding the Michaelis–Menten constant (K_M_), enzymes XI15 and XI13, with K_M_ 16.25 and 35.71 mM, respectively, present approximately 3.44‐fold and 1.56‐fold more affinity for xylose than OrpXI (K_M_ = 55.87 mM), requiring a smaller amount of substrate to reach half the maximum reaction rate. It is noteworthy that XI15 has the third lowest K_M_ amongst all reported XIs. Interestingly, the XI with the lowest K_M_, from the Termite gut (K_M_ = 10.52 mM), also belongs to the phylum *Bacillota*. XI09 (K_M_ = 48 mM), XI12 (K_M_ = 41.9 mM), and XI11 (K_M_ = 59.97 mM), as well as the control OrpXI, have lower affinity, requiring higher substrate concentrations to reach a significant reaction rate.

The K_M_ range of the XIs reported as functional is variable; however, all new XIs present values ​​higher than that of Termite gut (K_M_ = 10.52 mM) [61], which has a high affinity for xylose, but significantly lower than the K_M_ of *Ruminococcus flavefaciens* (K_M_ = 117.1 mM) [53], which has a considerably high K_M_. Nevertheless, studies have indicated that strategies such as targeted mutagenesis and adaptive evolution can improve the kinetics and affinity of enzymes [15, 53]. These approaches, when applied, offer a promising path to optimize the efficiency of the XIs reported here.

### 3.6 Novel XIs Allow High Xylose Fermentation Efficiency

**FIGURE 6:**
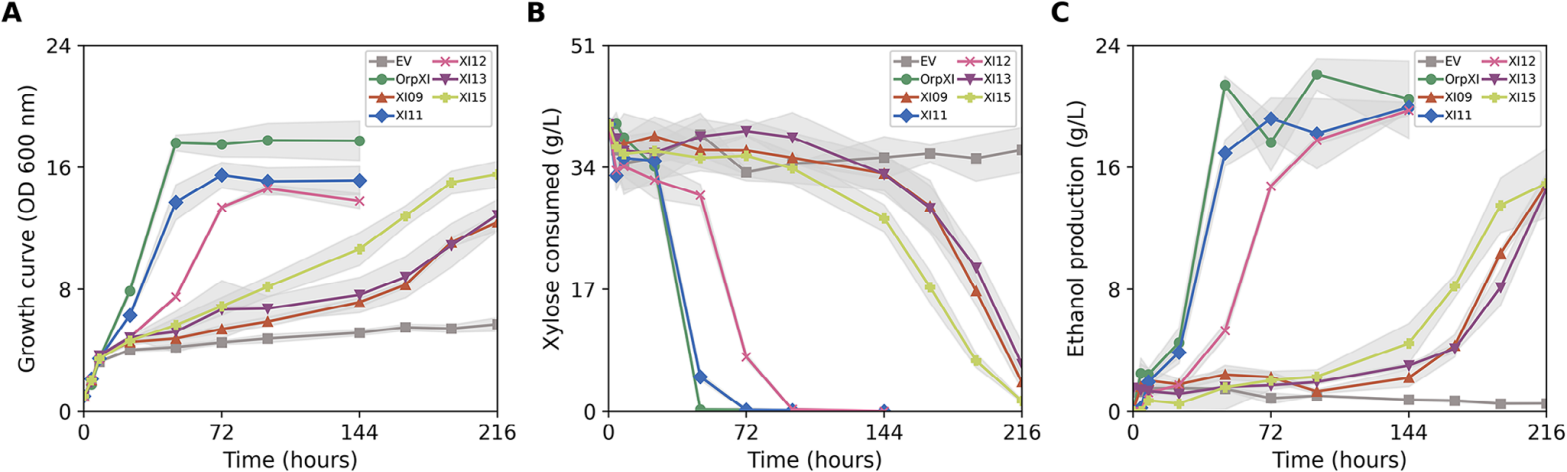
Batch semi‐anaerobic fermentation in xylose. Xylose fermentation was performed by strain BVY271 expressing different xylose isomerases under semi‐anaerobic conditions in YP medium containing 40 g/L xylose and 5% glucose. Legends represent the names of the tested samples, with the negative control shown as the empty vector (EV) and the positive control as *Orpinomyces sp*. ukk1 *xylA* (OrpXI) Panels represent cell growth (A), xylose consumption (B), and ethanol production (C) for 144 h (OrpXI, XI11, and XI12) or 216 h (EV, XI09, XI12, and XI15). Dark lines represent the sample data points, and shading represents the standard deviation in relation to the mean of the data. The experiment was performed in triplicate.

Fermentation assays were performed to evaluate whether enzymes XI09, XI11, XI12, XI13, and XI15 allowed the host strain to produce ethanol from xylose. The experiment was conducted in a complex medium, the best nutritional condition established in the previous results, supplemented with 40 g/L xylose and 5 g/L glucose. Glucose was added to the medium to allow faster initial growth of strains, favoring subsequent xylose metabolization, as previously reported [38]. Glucose was completely depleted within 8 h of fermentation in all samples analyzed (data not shown). As shown in Figure 6A, the strain not expressing an XI (EV) had the worst growth among the tested strains, reaching a final OD_600_ of 5.7 ± 0.36. In contrast, the strain expressing OrpXI exhibited the highest growth, displaying OD_600_ of 17.73 ± 1.04 after 144 h. The strains expressing the novel XI11 and XI12 achieved an OD_600_ of 15.1 ± 0.75 and 13.79 ± 0.40, respectively, in the same period. XI09, XI13, and XI15 presented slower growth, similar to that observed in microculture, and after 216 hours they reached OD_600_ of 12.39 ± 0.43, 12.86 ± 0.82, and 15.54 ± 0.70, respectively.

Xylose consumption and ethanol production (Figure 6B,C) greatly highlight the potential of the XI reported here. As expected, the host strain expressing EV was not able to metabolize xylose and produce ethanol. On the other hand, cells expressing OrpXI depleted 100% of xylose in 48 h and obtained an ethanol yield per gram of xylose (g/g) of 0.46 ± 0.05 in 144 h of fermentation, which represents 90% of the theoretical production. The five novel XIs evaluated were able to metabolize xylose and produce significant amounts of ethanol. Enzymes XI09, XI13, and XI15 promoted slower pentose conversions, resulting in the presence of residual xylose. The conversion values ​​for these enzymes were 89.59%, 83.67%, and 96.08%, respectively, after 216 h of fermentation. In contrast, enzymes XI11 and XI12 demonstrated faster xylose metabolism, depleting 100% of xylose in 72 and 96 h, respectively. Regarding ethanol yields, the strains expressing enzymes XI09, XI13, and XI15 obtained values of 0.38 ± 0.02 g/g, 0.43 ± 0.04 g/g, and 0.36 ± 0.04 g/g, respectively, in 216 h of fermentation. Meanwhile, enzymes XI11 and XI12 resulted in even higher ethanol yields: 0.46 ± 0.06 g/g and 0.45 ± 0.03 g/g, respectively, in 144 h of fermentation, achieving 90% and 88% of theoretical ethanol production—values ​​comparable to those obtained by the positive control OrpXI (90%).

The fermentation results of the present study are compared to other natural or codon‐optimized XIs previously expressed in *S. cerevisiae* in Table 2. For the parallel, only results obtained from strains that did not undergo adaptive laboratory evolution are shown. Notably, enzymes XI11 and XI12 here presented achieved ethanol yields much higher than those observed in other reported XIs [15, 20]. Only a few XIs described in the literature exhibit the ability to efficiently consume high levels of xylose when expressed in non‐evolved strains, making the results obtained particularly significant. Interestingly, despite the slow sugar conversion of enzymes XI09, XI13, and XI15, in comparison to XI11 and XI12, the ethanol yield values obtained with such enzymes ​​were comparable to those obtained by other XIs already reported in the literature: *P. ruminicola* (0.35 g/g) [15], *Bacteroides stercoris* (0.31 g/g) [62] and the fungi *Piromyces sp*. E2 (0.39 g/g) [17] and *Orpinomyces sp*. ukk1 (0.39 g/g) [16].

**TABLE 2:** Fermentation performance of different xylose isomerases expressed in *S. cerevisiae*.

Xylose isomerase	Conditions	Xylose conversion (%)	Ethanol (g/L)	Ethanol yield (g/g)	Reference
***Piromyces sp*. E2** ^c^	Anaerobic batch in chemostat, 2% glucose and 1% xylose	20	8.35	0.39	[17]
***Orpinomyces sp*. ukk1**	Anaerobic batch, 5% xylose	77.75	6.05	0.39	[16]
***Bacteroides stercoris***	Anaerobic batch, 2% xylose	80	4.9	0.31	[62]
***Prevotella ruminicola***	Anaerobic batch, 5% xylose	23.4	4.1	0.35	[15]
***Orpinomyces sp*. ukk1 (OrpXI)** ^a^, ^c^	Anaerobic batch, 0.5% glucose and 4% xylose	100	20.20 ± 2.06	0.46 ± 0.05	This study
**XI09 Putative *Bacilli bacterium*** ^b^, ^c^	Anaerobic batch, 0.5% glucose and 4% xylose	89.59 ± 0.78	14.86 ± 0.60	0.38 ± 0.02	This study
**XI11 Putative *Bacilli bacterium*** ^a^, ^c^	Anaerobic batch, 0.5% glucose and 4% xylose	100	19.95 ± 0.25	0.46 ± 0.01	This study
**XI12 Putative *Bacilli bacterium*** ^a^, ^c^	Anaerobic batch, 0.5% glucose and 4% xylose	100	19.73 ± 0.26	0.45 ± 0.01	This study
**XI13 Putative *Acholeplasmatales bacterium*** ^b^, ^c^	Anaerobic batch, 0.5% glucose and 4% xylose	83.67 ± 2.3	14.58 ± 0.56	0.43 ± 0.04	This study
**XI15 Putative *Closdridia bacterium*** ^b^, ^c^	Anaerobic batch, 0.5% glucose and 4% xylose	96.08 ± 0.43	14.94 ± 1.84	0.36 ± 0.04	This study

^a^ Sample data evaluated at 144 h of fermentation.

^b^ Sample data evaluated at 216 h of fermentation.

^c^ Ethanol yield calculations were based on the amount of sugar consumed, considering both glucose and xylose.

Comparison of enzymes XI13 and XI15 with XI11 and XI12, all expressed in the same strain, reveals substantial differences in the efficiency of xylose conversion to ethanol under fermentative conditions. Although XI13 presents a lower K_M_ (35.71 mM) compared to XI11 (59.97 mM) and XI12 (41.9 mM), indicating a higher affinity for xylose, its conversion rate is considerably slower, being unable to completely deplete xylose even after 216 h. In contrast, enzymes XI11 and XI12 are able to consume all xylose in 72 and 96 h, respectively. The difference in conversion efficiency between the enzymes may be related to intrinsic characteristics, such as lower structural stability at physiological pH or dependence on insufficiently available cofactors during fermentation [63]. Notably, XI13 exhibits lower enzymatic activity (0.0732 U·mg⁻¹) compared to XI11 (0.0861 U·mg⁻¹) and XI12 (0.0864 U·mg⁻¹), which likely contributes to its reduced capacity for xylose conversion under fermentative conditions. Curiously, although XI15 presents a lower K_M_ (16.25 mM) and relatively low enzymatic activity (0.0264 U·mg⁻¹), its performance in terms of xylose consumption and ethanol yield is slightly higher than that of XI13, but still lower than that observed for XI11 and XI12. However, more in‐depth biochemical and physiological studies are needed to elucidate the mechanisms underlying the divergences between the enzymatic profile and the performance observed under the fermentative conditions of the enzymes tested.

In XI identification studies, it is common to employ strategies such as mutagenesis and adaptive laboratory evolution to improve enzyme stability and substrate affinity, since most XIs show slow xylose conversion when functionally expressed in *S. cerevisiae* [19, 20, 55]. Lee et al. [55] increased the enzymatic activity of XI from *Piromyces sp*. E2 by 77% after three rounds of random mutation [55]. On the other hand, Peng et al. [20] improved the xylose conversion of XI isolated from *Bacteroides vulgatus* through adaptive evolution, increasing the conversion from 71.42% to 92.13% in 56 h of fermentation [20]. More recently, Chen et al. [19] also applied adaptive evolution strategies to improve enzymes from *Bacterium J10*, *Hallella seregens*, *Acetanaerobacterium elongatum*, and *Streptobacillus canis*, reducing the metabolization time of 40 g/L xylose to 16–18 h [19]. These approaches can be explored to optimize the conversion speed of substrates in the identified enzymes in this study, enhancing even further their application in biotechnological and industrial processes.

## 4 Conclusion

The use of omics tools, such as metagenomics and metatranscriptomics, applied under coherent conditions, such as herbivorous rumen within anaerobic conditions, figures as a promising strategy for new functional XIs sequences screening. This approach, combined with synthetic biology, has provided significant advances in the identification and characterization of enzymes of industrial interest. In this study, the combination of these approaches allowed the identification of seven putative XIs sequences from the rumen microbiota of herbivorous mammals. Surprisingly, all seven enzymes belonged to microorganisms of the phylum *Bacillota*, but only five of them showed functional activity when expressed in the yeast *S. cerevisiae*. Among these, three sequences were prospected from sheep rumen (XI11, XI12, and XI13), while the other two are from the cow (XI09) and camel (XI15) rumen. Although some of the XIs presented a slower conversion of xylose to xylulose, all enzymes were able to generate ethanol as a final product. However, sequences XI11 and XI12 stood out for their high fermentative capacity, being able to deplete 100% xylose in 72 and 96 h of fermentation, respectively, with remarkably high ethanol yields, without the need for further enzyme improvements. Fermentation efficiency of novel XIs is comparable to a very limited set of other sequences that have been previously described, while being tested in a non‐evolved strain. These results highlight the potential of omics tools in the identification of new enzymes, in addition to paving future advances in industrial xylose fermentation processes.

## Author Contributions

**Beatriz de Oliveira Vargas**: Writing–draft, writing–review and editing, validation, visualization, methodology, investigation, data curation, formal analysis. **Marcelo Falsarella Carazzolle**: Writing–review and editing, supervision, methodology, investigation. **Juliana Pimentel Galhardo**: Writing–review and editing, methodology, investigation, visualization. **Juliana José**: Writing–review, methodology, investigation, visualization. **Brenda Cristina de Souza**, **Jéssica Batista de Lima Correia**, and **Jade Ribeiro dos Santos**: Methodology, writing–review. **Fellipe da Silveira Bezerra de Mello**: Writing–draft, writing–review and editing, visualization, supervision, data curation, formal analysis. **Gonçalo Amarante Guimarães Pereira**: Writing–review and editing, project administration, funding acquisition.

## Conflicts of Interest

The authors declare no conflict of interest related to this work.

## Supporting information




**Supporting Information file 1**: biot70050‐sup‐0001‐SuppMat.docx

**Supporting Information file 2**: biot70050‐sup‐0002‐TableS7‐XI.xlsx

## Data Availability

The data underlying this article will be shared at reasonable request to the corresponding author.

## References

[1] S. Achinas and G. J. W. Euverink , “Consolidated Briefing of Biochemical Ethanol Production From Lignocellulosic Biomass,” Electronic Journal of Biotechnology 23 (2016): 44–53, 10.1016/j.ejbt.2016.07.006.

[2] H. Zabed , J. N. Sahu , A. N. Boyce , and G. Faruq , “Fuel Ethanol Production From Lignocellulosic Biomass: An Overview on Feedstocks and Technological Approaches,” Renewable and Sustainable Energy Reviews 66 (2016): 751–774, 10.1016/j.rser.2016.08.038.

[3] A. Lachke , “Biofuel From D‐Xylose—The Second Most Abundant Sugar,” Resonance 7 (2002): 50–58, 10.1007/BF02836736.

[4] J. T. Cunha , A. Romaní , C. E. Costa , I. Sá‐Correia , and L. Domingues , “Molecular and Physiological Basis of *Saccharomyces Cerevisiae* Tolerance to Adverse Lignocellulose‐Based Process Conditions,” Applied Microbiology and Biotechnology 103 (2019): 159–175, 10.1007/s00253-018-9478-3. 30397768

[5] M. M. Demeke , H. Dietz , Y. Li , et al., “Development of a D‐Xylose Fermenting and Inhibitor Tolerant Industrial *Saccharomyces Cerevisiae* Strain With High Performance in Lignocellulose Hydrolysates Using Metabolic and Evolutionary Engineering,” Biotechnology for Biofuels 6 (2013): 89, 10.1186/1754-6834-6-89. 23800147 PMC3698012

[6] M. A. Patiño , J. P. Ortiz , M. Velásquez , and B. U. Stambuk , “d ‐Xylose Consumption by Nonrecombinant *Saccharomyces cerevisiae*: A Review,” Yeast 36 (2019): 541–556, 10.1002/yea.3429. 31254359

[7] D. Brat , E. Boles , and B. Wiedemann , “Functional Expression of a Bacterial Xylose Isomerase in *Saccharomyces cerevisiae* ,” Applied and environmental microbiology 75 (2009): 2304–2311, 10.1128/AEM.02522-08. 19218403 PMC2675233

[8] N. W. Y. Ho , Z. Chen , and A. P. Brainard , “Genetically Engineered *Saccharomyces* Yeast Capable of Effective Cofermentation of Glucose and Xylose,” Applied and Environmental Microbiology 64 (1998): 1852–1859, 10.1128/aem.64.5.1852-1859.1998. 9572962 PMC106241

[9] P. Hoang Nguyen Tran , J. K. Ko , G. Gong , Y. Um , and S.‐M. Lee , “Improved Simultaneous Co‐Fermentation of Glucose and Xylose by *Saccharomyces cerevisiae* for Efficient Lignocellulosic Biorefinery,” Biotechnology for Biofuels 13 (2020): 12, 10.1186/s13068-019-1641-2. 31993090 PMC6975041

[10] B. Temer , L. V. Santos , V. A. Negri , et al., “Conversion of an Inactive Xylose Isomerase Into a Functional Enzyme by Co‐Expression of GroEL‐GroES Chaperonins in *Saccharomyces cerevisiae* ,” BMC Biotechnology 17 (2017): 11, 10.1186/s12896-017-0389-7. 28888227 PMC5591498

[11] R. M. Hochster and R. W. Watson , “Xylose Isomerase 1,” Journal of the American Chemical Society 75 (1953): 3284–3285, 10.1021/ja01109a516.

[12] C. J. Moes , I. S. Pretorius , and W. H. van Zyl , “Cloning and Expression of the *Clostridium thermosulfurogenes* D‐Xylose Isomerase Gene (xyLA) in *Saccharomyces cerevisiae* ,” Biotechnology Letters 18 (1996): 269–274, 10.1007/BF00142943.

[13] A. V. Sarthy , B. L. McConaughy , Z. Lobo , J. A. Sundstrom , C. E. Furlong , and B. D. Hall , “Expression of the *Escherichia coli* Xylose Isomerase Gene in *Saccharomyces cerevisiae* ,” Applied and Environmental Microbiology 53 (1987): 1996–2000, 10.1128/aem.53.9.1996-2000.1987. 2823706 PMC204047

[14] M. Walfridsson , X. Bao , M. Anderlund , G. Lilius , L. Bülow , and B. Hahn‐Hägerdal , “Ethanolic Fermentation of Xylose With *Saccharomyces cerevisiae* Harboring the *Thermus thermophilus* xylA gene, which expresses an active xylose (glucose) isomerase,” Applied and Environmental Microbiology 62 (1996): 4648–4651, 10.1128/aem.62.12.4648-4651.1996. 8953736 PMC168291

[15] R. E. Hector , B. S. Dien , M. A. Cotta , and J. A. Mertens , “Growth and Fermentation of D‐Xylose by *Saccharomyces cerevisiae* Expressing a Novel D‐Xylose Isomerase Originating From the Bacterium *Prevotella ruminicola* TC2‐24,” Biotechnology for Biofuels 6 (2013): 84, 10.1186/1754-6834-6-84. 23721368 PMC3673840

[16] A. Madhavan , S. Tamalampudi , K. Ushida , et al., “Xylose Isomerase From Polycentric Fungus *Orpinomyces*: Gene Sequencing, Cloning, and Expression in *Saccharomyces cerevisiae* for Bioconversion of Xylose to Ethanol,” Applied Microbiology and Biotechnology 82 (2009): 1067–1078, 10.1007/s00253-008-1794-6. 19050860

[17] M. Kuyper , H. Harhangi , A. Stave , et al., “High‐Level Functional Expression of a Fungal Xylose Isomerase: The Key to Efficient Ethanolic Fermentation of Xylose by *Saccharomyces cerevisiae*?,” FEMS Yeast Research 4 (2003): 69–78, 10.1016/S1567-1356(03)00141-7. 14554198

[18] M. Kuyper , M. M. P. Hartog , M. J. Toirkens , et al., “Metabolic Engineering of a Xylose‐Isomerase‐Expressing Strain for Rapid Anaerobic Xylose Fermentation,” FEMS Yeast Research 5 (2005): 399–409, 10.1016/j.femsyr.2004.09.010. 15691745

[19] S. Chen , Z. Xu , B. Ding , et al., “Big Data Mining, Rational Modification, and Ancestral Sequence Reconstruction Inferred Multiple Xylose Isomerases For Biorefinery,” Science Advances 9 (2023): add8835, 10.1126/sciadv.add8835. PMC9891696 36724227

[20] B. Peng , S. Huang , T. Liu , and A. Geng , “Bacterial Xylose Isomerases From the Mammal Gut Bacteroidetes Cluster Function in *Saccharomyces cerevisiae* for Effective Xylose Fermentation,” Microbial Cell Factories 14 (2015): 70, 10.1186/s12934-015-0253-1. 25981595 PMC4436767

[21] B. A. Dehority , “Mechanism of Isolated Hemicellulose and Xylan Degradation by Cellulolytic Rumen Bacteria,” Applied Microbiology 16 (1968): 781–786, 10.1128/am.16.5.781-786.1968. 5690671 PMC547517

[22] J. Handelsman , “Metagenomics: Application of Genomics to Uncultured Microorganisms,” Metagenomics: Application of Genomics to Uncultured Microorganisms 68 (2004): 669–685, 10.1128/MBR.68.4.669-685.2004. PMC539003 15590779

[23] P. J. Weimer , “Degradation of Cellulose and Hemicellulose by Ruminal Microorganisms,” Microorganisms 10 (2022): 2345, 10.3390/microorganisms10122345. 36557598 PMC9785684

[24] J. Hou , Y. Shen , C. Jiao , R. Ge , X. Zhang , and X. Bao , “Characterization and Evolution of Xylose Isomerase Screened From the Bovine Rumen Metagenome in *Saccharomyces cerevisiae* ,” Journal of Bioscience and Bioengineering 121 (2016): 160–165, 10.1016/j.jbiosc.2015.05.014. 26160406

[25] N. Parachin and M. F. Gorwa‐Grauslund , “Isolation of Xylose Isomerases by Sequence‐ and Function‐Based Screening From a Soil Metagenomic Library,” Biotechnology for Biofuels 4 (2011): 9, 10.1186/1754-6834-4-9. 21545702 PMC3113934

[26] P. C. Silva , J. A. Ceja‐Navarro , F. Azevedo , U. Karaoz , E. L. Brodie , and B. Johansson , “A Novel d‐Xylose Isomerase From the Gut of the Wood Feeding Beetle *Odontotaenius disjunctus* Efficiently Expressed in *Saccharomyces cerevisiae* ,” Scientific Reports 11 (2021): 4766, 10.1038/s41598-021-83937-z. 33637780 PMC7910561

[27] A. Madhavan , R. Sindhu , B. Parameswaran , R. K. Sukumaran , and A. Pandey , “Metagenome Analysis: A Powerful Tool for Enzyme Bioprospecting,” Applied Biochemistry and Biotechnology 183 (2017): 636–651, 10.1007/s12010-017-2568-3. 28815469

[28] F. Warnecke and M. Hess , “A perspective: Metatranscriptomics as a Tool for the Discovery of Novel Biocatalysts,” Journal of Biotechnology 142 (2009): 91–95, 10.1016/j.jbiotec.2009.03.022. 19480952

[29] J. Mistry , R. D. Finn , S. R. Eddy , A. Bateman , and M. Punta , “Challenges in Homology Search: HMMER3 and Convergent Evolution of Coiled‐Coil Regions,” Nucleic Acids Res. 41 (2013): 121, 10.1093/nar/gkt263. PMC3695513 23598997

[30] L. Fu , B. Niu , Z. Zhu , S. Wu , and W. Li , “CD‐HIT: Accelerated for Clustering the Next‐Generation Sequencing Data,” Bioinformatics 28 (2012): 3150–3152, 10.1093/bioinformatics/bts565. 23060610 PMC3516142

[31] B. Q. Minh , H. A. Schmidt , O. Chernomor , et al., “IQ‐TREE 2: New Models and Efficient Methods for Phylogenetic Inference in the Genomic Era,” Molecular biology and evolution 37 (2020): 1530–1534, 10.1093/molbev/msaa015. 32011700 PMC7182206

[32] K. D. Yamada , K. Tomii , and K. Katoh , “Application of the MAFFT sequence Alignment Program to Large Data—Reexamination of the Usefulness of Chained Guide Trees,” Bioinformatics 32 (2016): 3246–3251, 10.1093/bioinformatics/btw412. 27378296 PMC5079479

[33] K. Katoh , K. Kuma , H. Toh , and T. Miyata , “MAFFT Version 5: Improvement in Accuracy of Multiple Sequence Alignment,” Nucleic Acids Research 33 (2005): 511–518, 10.1093/nar/gki198. 15661851 PMC548345

[34] T. W. Christianson , R. S. Sikorski , M. Dante , J. H. Shero , and P. Hieter , “Multifunctional Yeast High‐Copy‐Number Shuttle Vectors,” Gene 110 (1992): 119–122, 10.1016/0378-1119(92)90454-W. 1544568

[35] W. J. Dower , J. F. Miller , and C. W. Ragsdale , “High Efficiency Transformation of *E. coli* by High Voltage Electroporation,” Nucleic Acids Research 16 (1988): 6127–6145, 10.1093/nar/16.13.6127. 3041370 PMC336852

[36] H. C. Birnboim and J. Doly , “A Rapid Alkaline Extraction Procedure for Screening Recombinant Plasmid DNA,” Nucleic Acids Research 7 (1979): 1513–1523, 10.1093/nar/7.6.1513. 388356 PMC342324

[37] L. C. Basso , H. V. De Amorim , A. J. De Oliveira , and M. L. Lopes , “Yeast Selection for Fuel Ethanol Production in Brazil,” FEMS Yeast Research 8 (2008): 1155–1163, 10.1111/j.1567-1364.2008.00428.x. 18752628

[38] L. V. Dos Santos , M. F. Carazzolle , S. T. Nagamatsu , et al., “Unraveling the Genetic Basis of Xylose Consumption in Engineered *Saccharomyces cerevisiae* Strains,” Scientific Reports 6 (2016): 38676, 10.1038/srep38676. 28000736 PMC5175268

[39] A. L. Goldstein and J. H. McCusker , “Three New Dominant Drug Resistance Cassettes for Gene Disruption in *Saccharomyces cerevisiae* ,” Yeast 15 (1999): 1541–1553, 10.1002/(SICI)1097-0061(199910)15:14<1541::AID-YEA476>3.0.CO;2-K. 10514571

[40] F. D. S. B. De Mello , C. Maneira , F. U. L. Suarez , et al., “Rational Engineering of Industrial *S. cerevisiae*: Towards Xylitol Production From Sugarcane Straw,” Journal, Genetic Engineering & Biotechnology 20 (2022): 80, 10.1186/s43141-022-00359-8. PMC9133290 35612634

[41] R. D. Gietz and R. H. Schiestl , “High‐Efficiency Yeast Transformation Using the LiAc/SS Carrier DNA/PEG Method,” Nature Protocols 2 (2007): 31–34, 10.1038/nprot.2007.13. 17401334

[42] M. Lõoke , K. Kristjuhan , and A. Kristjuhan , “Extraction of Genomic DNA From Yeasts for PCR‐Based Applications,” Biotechniques 50 (2011): 325–328, 10.2144/000113672. 21548894 PMC3182553

[43] F. De Mello S. B. da , A. L. V. Coradini , et al., “Static Microplate Fermentation and Automated Growth Analysis Approaches Identified a Highly‐Aldehyde Resistant *Saccharomyces cerevisiae* Strain,” Biomass and Bioenergy 120 (2019): 49–58, 10.1016/j.biombioe.2018.10.019.

[44] C. R. Harris , K. J. Millman , S. J. Van Der Walt , et al., “Array programming With NumPy,” Nature 585 (2020): 357–362, 10.1038/s41586-020-2649-2. 32939066 PMC7759461

[45] J. D. Hunter , “Matplotlib: A 2D Graphics Environment,” Computing in Science & Engineering 9 (2007): 90–95, 10.1109/MCSE.2007.55.

[46] W. McKinney , “Data Structures for Statistical Computing in Python,” in *Proceedings of the 9th Python in Science Conference* (SciPy, 2010), 56–61, 10.25080/Majora-92bf1922-00a.

[47] T. E. Oliphant , Guide to NumPy, 1st ed. (Trelgol Publishing, 2006).

[48] R. Tang , P. Ye , H. S. Alper , Z. Liu , X. Zhao , and F. Bai , “Identification and Characterization of Novel Xylose Isomerases From a Bos Taurus Fecal Metagenome,” Applied Microbiology and Biotechnology 103 (2019): 9465–9477, 10.1007/s00253-019-10161-1. 31701197

[49] L. S. Parreiras , R. J. Breuer , R. A. Narasimhan , et al., “Engineering and Two‐Stage Evolution of a Lignocellulosic Hydrolysate‐Tolerant *Saccharomyces cerevisiae* Strain for Anaerobic Fermentation of Xylose From AFEX Pretreated Corn Stover,” PLoS One 9 (2014): 107499, 10.1371/journal.pone.0107499. PMC4164640 25222864

[50] B. D. O. Vargas , J. R. Dos Santos , G. A. G. Pereira , and F. D. S. B. De Mello , “An Atlas of Rational Genetic Engineering Strategies for Improved Xylose Metabolism in *Saccharomyces cerevisiae* ,” PeerJ 11 (2023): 16340, 10.7717/peerj.16340. PMC10691383 38047029

[51] R. Amore , M. Wilhelm , and C. P. Hollenberg , “The Fermentation of Xylose ?An Analysis of the Expression of *Bacillus* and *Actinoplanes* Xylose Isomerase Genes in Yeast,” Applied Microbiology and Biotechnology 30 (1989): 351–357, 10.1007/BF00296623.

[52] B. Hahn‐Hägerdal , C. F. Wahlbom , M. Gárdonyi , W. H. Van Zyl , R. R. C. Otero , and L. J. Jönsson , “Metabolic Engineering of *Saccharomyces cerevisiae* for Xylose Utilization,” Advances in Biochemical Engineering/Biotechnology 73 (2001): 53–84, 10.1007/3-540-45300-8_4. Metab. Eng.. 11816812

[53] K. A. Aeling , K. A. Salmon , J. M. Laplaza , et al., “Co‐Fermentation of Xylose and Cellobiose by an Engineered *Saccharomyces cerevisiae* ,” Journal of Industrial Microbiology & Biotechnology 39 (2012): 1597–1604, 10.1007/s10295-012-1169-y. 22911235

[54] B. Hahn‐Hägerdal , K. Karhumaa , C. U. Larsson , M. Gorwa‐Grauslund , J. Görgens , and W. H. Van Zyl , “Role of Cultivation Media in the Development of Yeast Strains for Large Scale Industrial Use,” Microbial Cell Factories 4 (2005): 31, 10.1186/1475-2859-4-31. 16283927 PMC1316877

[55] S. M. Lee , T. Jellison , and H. S. Alper , “Directed Evolution of Xylose Isomerase for Improved Xylose Catabolism and Fermentation in the Yeast *Saccharomyces cerevisiae* ,” Applied and Environmental Microbiology 78 (2012): 5708–5716, 10.1128/AEM.01419-12. 22685138 PMC3406111

[56] M. Fuxreiter , Z. Böcskei , A. Szeibert , et al., “Role of Electrostatics at the Catalytic Metal Binding Site in Xylose Isomerase Action: Ca2+‐Inhibition and Metal Competence in the Double Mutant D254E/D256E,” Proteins: Structure, Function and Genetics 28 (1997): 183–193, 10.1002/(SICI)1097-0134(199706)28:2<183::AID-PROT7>3.0.CO;2-G. 9188736

[57] M. Lee , H. J. Rozeboom , P. P. De Waal , R. M. De Jong , H. M. Dudek , and D. B. Janssen , “Metal Dependence of the Xylose Isomerase From *Piromyces* sp. E2 Explored by Activity Profiling and Protein Crystallography,” Biochemistry 56 (2017): 5991–6005, 10.1021/acs.biochem.7b00777. 29045784 PMC5688467

[58] H. Van Tilbeurgh , J. Jenkins , and M. Chiadmi , “Protein Engineering of Xylose (Glucose) Isomerase From *Actinoplanes missouriensis*. 3. Changing Metal Specificity and the pH Profile by Site‐Directed Mutagenesis,” Biochemistry 31 (1992): 5467–5471, 10.1021/bi00139a007. 1610793

[59] P. B. M. Van Bastelaere , M. Callens , W. A. E. Vangrysperre , and H. L. M. Kersters‐Hilderson , “Binding Characteristics of Mn2+, Co2+ and Mg2+ Ions With Several D‐Xylose Isomerases,” Biochemical Journal 286 (1992): 729–735, 10.1042/bj2860729. 1417732 PMC1132964

[60] S. M. Gaikwad , M. B. Rao , and V. V. Deshpande , “D‐Glucose/Xyiose Isomerase From Streptomyces: Differential Roles of Magnesium and Cobalt Ions,” Enzyme and Microbial Technology 14 (1992): 317–320, 10.1016/0141-0229(92)90158-K.

[61] S. Katahira , N. Muramoto , S. Moriya , et al., “Screening and Evolution of a Novel Protist Xylose Isomerase From the Termite *Reticulitermes speratus* For Efficient Xylose Fermentation in *Saccharomyces cerevisiae* ,” Biotechnology for Biofuels 10 (2017): 203, 10.1186/s13068-017-0890-1. 28852424 PMC5569483

[62] S. J. Ha , S. R. Kim , J. H. Choi , M. S. Park , and Y. S. Jin , “Xylitol Does not Inhibit Xylose Fermentation by Engineered *Saccharomyces cerevisiae* Expressing xylA as Severely as it Inhibits Xylose Isomerase Reaction In Vitro,” Applied Microbiology and Biotechnology 92 (2011): 77–84, 10.1007/s00253-011-3345-9. 21655987

[63] J.‐H. Bae , M.‐J. Kim , B. H. Sung , Y.‐S. Jin , and J.‐H. Sohn , “Directed Evolution and Secretory Expression of Xylose Isomerase for Improved Utilisation of Xylose in *Saccharomyces cerevisiae* ,” Biotechnology for Biofuels 14 (2021): 223, 10.1186/s13068-021-02073-y. 34823570 PMC8613937

