# Supplementary material for: Engineering Saccharomyces Cerevisiae With Novel Functional Xylose Isomerases From Rumen Microbiota for Enhanced Biofuel Production
Source: Biotechnol J. 2025 Jun 9;20(6):e70050. doi: 10.1002/biot.70050 (PMC12149495; doi:10.1002/biot.70050)
Supplement: Supplementary file 1 — Supporting Information file 1: biot70050‐sup‐0001‐SuppMat.docx [file BIOT-20-e70050-s002.docx]

Supplementary Material Vargas *et al.* 2025

**Engineering *S. cerevisiae* with Novel Functional Xylose Isomerases from Rumen Microbiota for Enhanced Biofuel Production**

Beatriz de Oliveira Vargas^a^, Marcelo Falsarella Carazzolle^a^, Juliana Pimentel Galhardo^a^, Juliana José^a^, Brenda Cristina de Souza^a^, Jéssica Batista de Lima Correia^a^, Jade Ribeiro dos Santos^a^, Gonçalo Amarante Guimarães Pereira^a,*^, Fellipe da Silveira Bezerra de Mello^a^

^a^Departamento de Genética, Evolução, Microbiologia e Imunologia, UNICAMP, Campinas, SP, Brazil

^*^ Corresponding author:  [goncalo@unicamp.br](mailto:goncalo@unicamp.br)

**Supplementary Figures**

**Supplementary Figure S1**


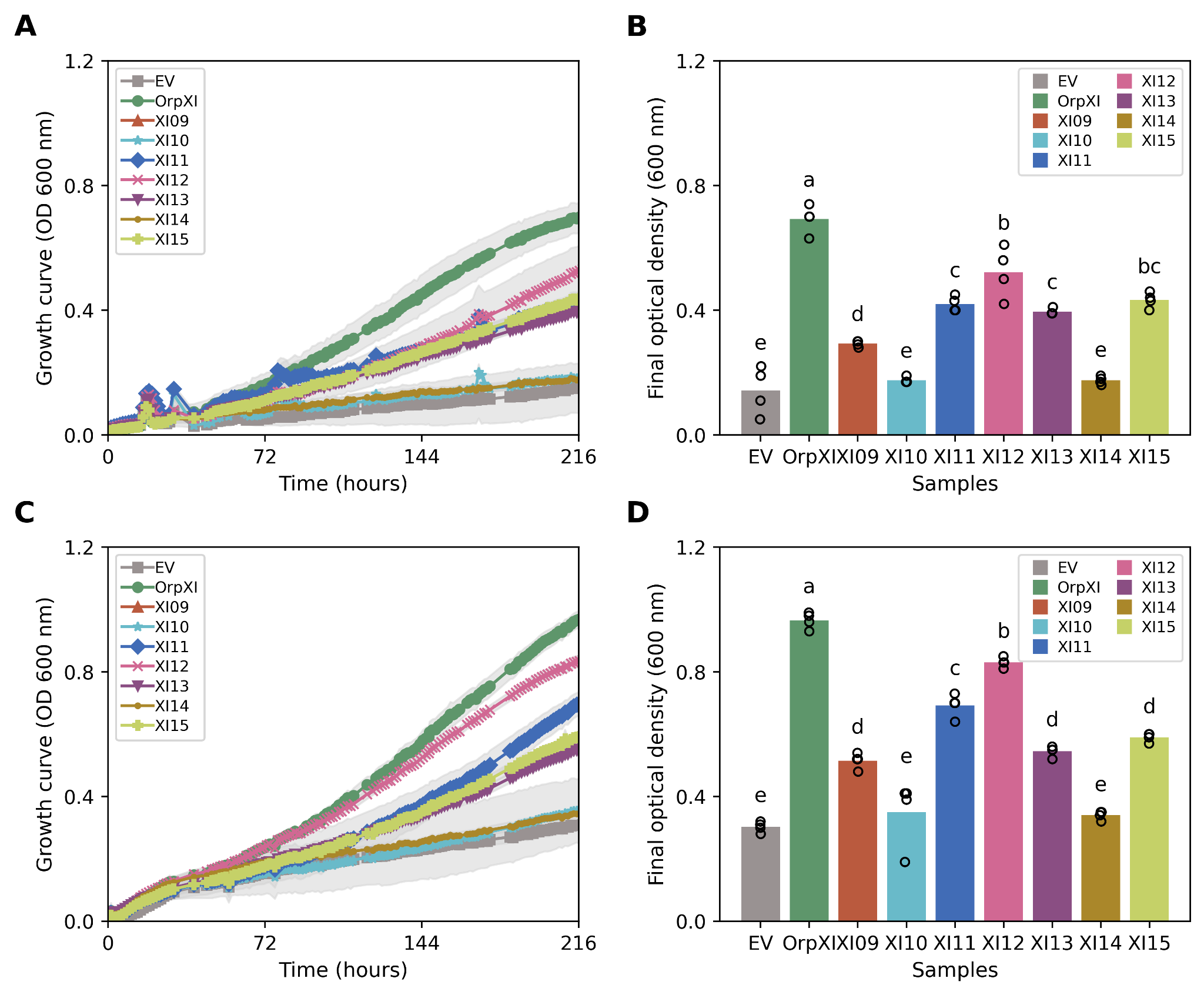


**Fig. S1**. Growth analysis in microculture of *Saccharomyces cerevisiae* GGY018 expressing various xylose isomerases identified through metagenomic and metatranscriptomic analysis of the rumen microbiota of herbivorous mammals. Growth comparison under conditions of varying nutrient availability, using xylose as the sole carbon source. (A) Growth curve in minimal medium containing 5% xylose (YNBX) over 216 hours. (B) Final optical density (OD_600_) after 216 hours of growth in YNBX medium. (C) Growth curve in rich medium containing 5% xylose (YPX) over 216 hours. (D) Final OD_600_ after 216 hours of growth in YPX medium. The figure legends represent the names of the tested samples, with the negative control shown as the empty vector (EV) and the positive control as Orpinomyces sp. *xylA* (OrpXI). Shading in panels (A) and (C) represents the standard deviation of the sample mean. In panels (B) and (D), different letters denote statistically significant (p<0.05) differences between the samples for each evaluated parameter, as determined by ANOVA, and circles represent the data points. The experiment was performed in quadruplicate.


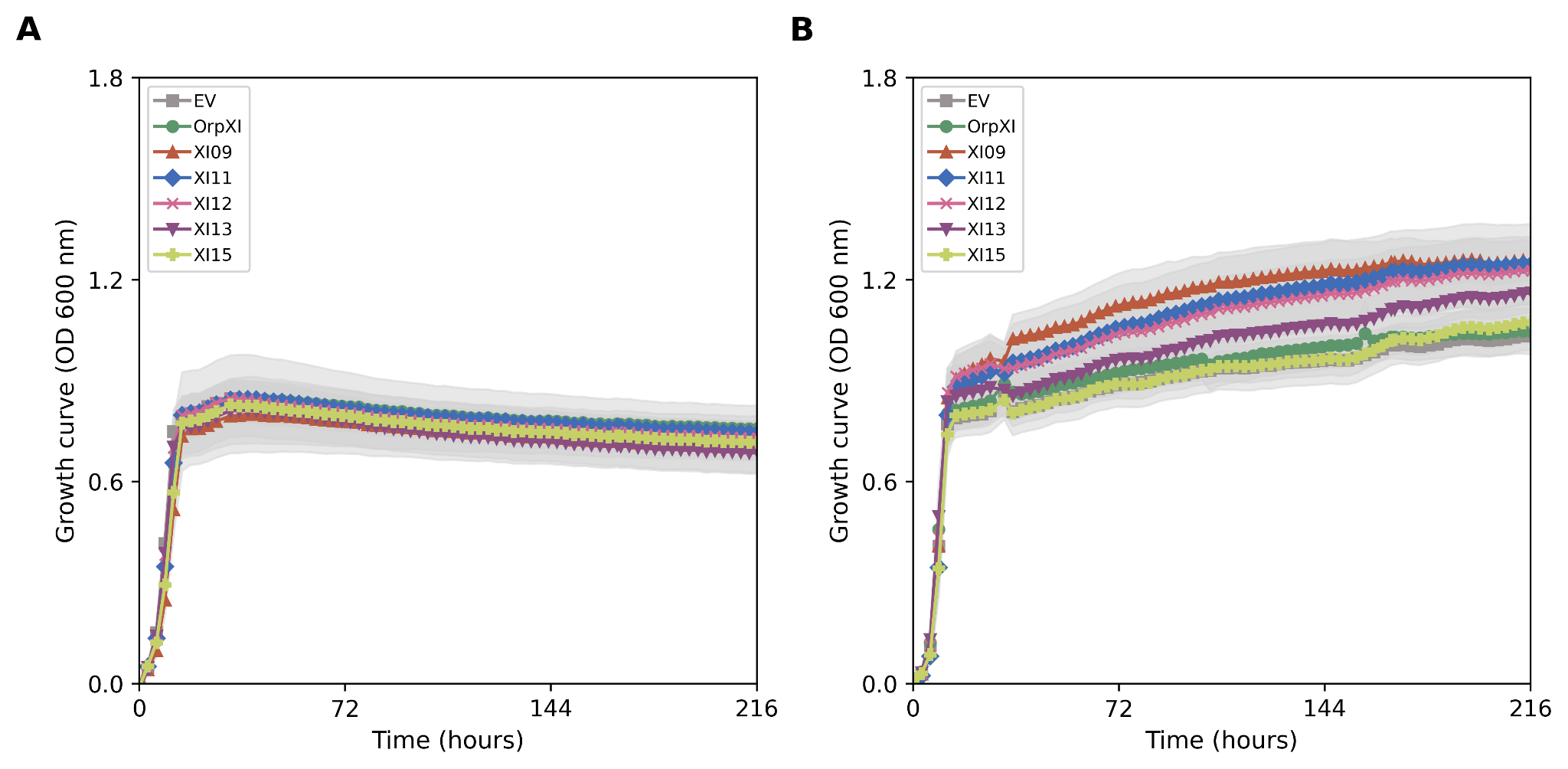


**Fig. S2**. Growth analysis in microculture of *Saccharomyces cerevisiae* BVY271 expressing various xylose isomerases identified through metagenomic and metatranscriptomic analysis of the rumen microbiota of herbivorous mammals. (A) Growth curve in minimal medium containing 2% glucose (YNB) over 216 hours. (B) Growth curve in rich medium containing 2% glucose (YPD) over 216 hours. The figure legends represent the names of the tested samples, with the negative control shown as the empty vector (EV) and the positive control as Orpinomyces sp. *xylA* (OrpXI). Shading represents the standard deviation of the sample mean. The experiment was performed in quintuplicate.

| **Table S1**. *Saccharomyces cerevisiae* strains and plasmids used in this study. | | |
| --- | --- | --- |
| **Strain** | **Relevant genotype** | **References** |
| **GGY018** | *Derived PE-2xSA-1; MATa; gre3Δ; CEN12::pTDH1-TAL1-tTDH1-pPGK1-RKI1-tPGK1; CEN13::pTDH1-TKL1-tTDH1-pPGK1-RPE1-tPGK1* | This study |
| **LVY27** | *Derived PE-2; MATα; CEN5::pTDH1-xylA-tTDH1; gre3Δ; CEN2::pADH1-XKS1-tADH1; CEN8::pADH1-XKS1-tADH1; CEN12::pTDH1-TAL1-tTDH1-pPGK1-RKI1-tPGK1; CEN13::pTDH1-TKL1-tTDH1-pPGK1-RPE1-tPGK1* | dos Santos et al., 2016 |
| **BVY270** | LVY27; *pTDH1-xylA-tTDH1Δ::pTEF1-hphMX6-tTEF1* | This study |
| **BVY271** | BVY270; *ura3Δ* | This study |
| **Plasmid** | **Relevant features** | **References** |
| **pAG32** | *HphMX6* - Hygromycin B resistance | Goldstein, AL & McCusker, 1999 |
| **pGS** | pTEF1-Cas9-tCYC1; pSNR52-sgRNA-tSUP4 | De Mello et al., 2022 |
| **pRS426_GPD** | *GPD1p_CYC1t; URA3;* 2μ | Christianson et al., 1992 |
| **p426_GPD_OrpXI** | *URA3*; *Orpinomyces sp.* *xylA* | This study |
| **p426_GPD_XI09** | *URA3*; XI09 *xylA* | This study |
| **p426_GPD_XI10** | *URA3*; XI10 *xylA* | This study |
| **p426_GPD_XI11** | *URA3*; XI11 *xylA* | This study |
| **p426_GPD_XI12** | *URA3*; XI12 *xylA* | This study |
| **p426_GPD_XI13** | *URA3*; XI13 *xylA* | This study |
| **p426_GPD_XI14** | *URA3*; XI14 *xylA* | This study |
| **p426_GPD_XI15** | *URA3*; XI15 *xylA* | This study |
|  |  |  |

| **Table S2**. Oligonucleotides used in this study | | |
| --- | --- | --- |
| **Name** | **Sequence 5’ to 3’** | **Description** |
| **URA3KO_F** | CAGAATAGCAGAATGGGCAGACATTACGA  ATGCACACGGTGTGGTGGGCTGATCA | Hybridization of the donor DNA for the *URA3* gene |
| **URA3KO_R** | CTTCTTCCGCCGCCTGCTTCAAACCGCTA  ACAATATGATCAGCCCACCACACCGT | Hybridization of the donor DNA for the *URA3* gene |
| **BVO_049** | ACAACGAAAGAGATCTAAATTTAGAAGACAG  CAAGACTAACGCGCCAGATCTGTTTAGCTT | Amplification with homology to CEN5 |
| **BVO_050** | CGATGCCGCTATAATGGAAAAAAAGGAGTCG  CGACATATGGCCGATTCATTAATGCAGGT | Amplification with homology to CEN5 |
| **BVO_051** | AAAAGTGGGATGCCTTGGTT | Check *hphMX6* to CEN5 |
| **BVO_015** | ATGACTAAAGAATATTTTCCAAC | Check *Orpinomyces sp.* *xylA* |
| **SCO_009** | TTCTCAAGCAAGGTTTTCAG | Check *p426_GPD* |
| **SCO_018** | TAAGTCTTTCGGTGTTGTTC | Check XI09 *xylA* |
| **SCO_019** | CATGGGAATCCGATGACAAG | Check XI10 *xylA* |
| **SCO_020** | GTTGACAAGGACAAGGAC | Check XI11 *xylA* |
| **SCO_021** | CAGCTACCATGAACAAGAAC | Check XI12 *xylA* |
| **SCO_022** | GATTACCAAAGACCCTATCG | Check XI13 *xylA* |
| **SCO_023** | GGTAACAAGGAAGCCATG | Check XI14 *xylA* |
| **SCO_024** | GGTTCATCTGGTATGGCC | Check XI15 *xylA* |
|  |  |  |

| **Table S3**. Amino acids sequences of the novel xylose isomerases presented in this study. | |
| --- | --- |
|  | |
| > XI09 Putative *Bacilli bacterium* MEYFNFVKKVVFEGPESKNPFAFKFYDPEKIILGKPMKEHLKFAMAWWHNLGADGTDMFGRGTIDKSFGVVPGTLEHAKAKVDAGFEFMTKMGIKYFCFHDVDLVPEQKDINDTNKMLDEVSDYILEKEKETGIKCLWGTANLFGNPRYMAGAGTSNRVDVYCFAAAQIKKALDITVKLGGENYVFWGGREGYETLLNTDMGLEEENIAQLMRMAVEYGRKIGLKGDFYIEPKPKEPMKHQYDFDAATSIGFLRKYGLDKDFKMNIEANHAELAKHTFEHELRVSRLNGMLGSIDANEGDCVLGWDVDRFPANVYTATFAMLEILKAGKLSGGLNFDAKTRRASNTYEDMFKAYILGMDTWALGLINAAKLIEDGRIEKFVADRYATWNSDLGKKIRAGKADLEELAKIGCDQKYFDAENSGREEELQEILNSVLFR | |
| > XI10 Putative *Acholeplasmatales bacterium*  MKEYFKIGKINYEGPKSNNPFSFKYYNADEVIMGKTMKEHLRFAMSWWHTLGAKGSDQFGEDSAVRPWESDDKIEEAFNKVDAGFEFMQKLGIEYFCFHDRDLAPEMNTLKESNEVLDKISDYILKKQKETGIKCLWGTANCFNNKRYALGASTSPNANAFAYAAAQIKKAMDVTKKLGGKNYVFWGGREGYDTLLNTNTKLELDHYAQMLRLASDYNEKSGLNAQLLIEPKPKEPTKHQYDFDVQTVLGFLHTYNLESKFKFNIEANHATLAGHTFQHELNMARINNALGSIDANQGDSLLGWDTDQFPTNIYDATLAMYEVLKMGGFSTGGLNFDAKVRRASFEEEDLFLAYISGMDTFAKGLRVAAKLLEDRVLEDFVANRYKSYNEGIGELINQGKTSFEELEKYALTHEFGKNESGRQEMLESILNSYILEK | |
| > XI11 Putative *Bacilli bacterium*  MEYFPFVKQVQYKGPSSTEPFAFKYYDANRIVCGKPMKEWMPFAMAWWHNLGAAGTDMFGGNTMDKSWGVDKDKDPMGYAKAKVDAGFEFMQKMGIEYYCFHDVDLVPECDDITVMYQRLDEIGEYLLKKQKETGIKLLWATANAFGHRRFMNGAGSSNSAEVYCFAAAQIKKALELCVKLGGKGYVFWGGREGYETLLNTDVKFEEENIANLMRVARDYGRKIGFKGDFYIEPKPKEPTKHQYDFDAATAIGFLRKYGLDKDFKMNIEANHATLAGHTFEHELRISAMNGMLGSIDANEGDTLLGWDVDRFPANVYSATFAMLEVIKAGGLTGGFNFDAKTRRASNTYEDMFKAYVLGMDTFALGLLNAEAIIKDGRIDKFVEDRYASYKSGIGAKIRDHSATIEDLAAHALETKVCPDPGSGDEEELQEILNQVMFGKK | |
| > XI12 Putative *Bacilli bacterium*  MEYFDFIGKVQYEGPKSKNPYAFKFYDANEVIMGKPMKDHLKFAMAWWHNLGAAGVDMFGPATMNKNFGAKPGTMEYAKAKVDAGFEFMKKLGIEYYCFHDVDLVPEQLDINETNKWLDELSDYILTKQKGTNIRCLWGTANNFGNKRYMNGAGSSNSVEVYCFAAAQIKKALDITVKLGGENYVFWGGREGYETLLNTDMGLEEENIAALMKMAVAYGRKIGLKGDFYIEPKPKEPMKHQYDFDAATSIGFLRKYGLDKDFKMNIEANHAELANHTFEHELRVSRINGMLGSIDANEGDPILGWDVDRFPANVYSATFAMYELLKSPGLTGGCNFDAKNRRASNTFEDMVKGYILGMDTFALGLRKAAKLIEDGRIEKFIKERYSSWNGELGKKIRAGKTTLEDLFNEACKMKKTDEPSSGKEEELQEILNTILFND | |
| > XI13 Putative *Acholeplasmatales bacterium*  MEYFDFSEIKYEGSLSKNPLSFKEYNKDEVILGKKMEDHLRFAISWWHTLSAFGSDPFGSNTMERAWITKDPIETAKNKIDAGFEFMQKLSIKYFCFHDRDLVEEGKDLAETNERLDIVSDYLLKKMKETGIKCLWGTANLFNNKRFMHGASTSPNADVFAYSCAQVKKALDITKKLNGENYVFWGGREGYETLLNTNTSMELDHFALFLKLAKDYANEIGFKGQFLIEPKPKEPTKHQYDFDTQTVLSFLRKYNLEKDFKVNIEANHATLAGHTFNHELNIARINNVLGSIDANQGDLLLGWDTDQFPTNVYDATLCMYEVLKNGGLNPGGLNFDSKVRRSSFENIDLAYAYIAGMDTFAKGLKVAAKLIEDKVFDNIIDERYKSYKTGIGKKINDKNITFKELNEYALTLKEIKNDSGHQELLESILNQYIYNTK | |
| > XI14 Putative *Lachnospiraceae bacterium*  MSEYFKNVPKVKYEGPKSKNPFAFKFYDPDRKVAGKTMREQLKFGMAWWHTLCANGQDMFGSPTMDKSFGNKEAMDLAKAKVDAGFEFMDKLGIDYYCFHDRDIAPEGRNIDESTENLNTIVDILKEKQKESGKKLLWGTANLFNNPRYMHGAGTAPNADAFAYAAAQLKAALEATKTLDGAGYTCWGGREGYETLLNTDMGLELDNMARLFKMLIAHADKIGFTGPFYIEPKPKEPTKHQYDFDAATCVNFLRAYGLMDRFRLNIETNHATLAGHTMQHELRVARVNGVFGSVDANQGDMLLGWDTDQFPTDVYGAGLAMYEILKAGGFTTGGLNFDAKQRRGSFTLDDVAIAHIAGMDGFALGLLLADRMIEDGRIDKFVEERYASWKSGIGKSIIDGKESLESLYAYVQKMGEVTTNTSGRQEELESVMNQCLMSIDF | |
| > XI15 Putative *Clostridia bacterium*  MYIDIDKIKYEGSKSRNPFAFRFYDKNRVIAGKTMGQHLKFAMSWWHTINASGTDMFGGDTVDKTLGSSGMAMYKAKADFAFEIMEKLGIDYYCFHDVDIAPEGETLAESVAYLNEMTDYLLEKQKKTGIRPLWVTANMFGAKKFMAGAATSPDADVFAISAGKVKAAIDAAVKLGAKGYVFWGGREGYDTLLNTDMDLELKNLGRFMKLARDYGRAHGFDGDFYIEPKPKEPTKHQYDFDAATCANFLRINGLENDFRLNIEANHATLAGHTFQHELRTATVNGLFGSIDANQGDMLLGWDTDQFPTNVYDTTLCMYEVIKAGGFTNGGLNFDAKARRQSNTLEDILLSYIAGMDAFALGLIKAYRIIDDGRIEKFINERYASYSKGIGLRITEGKETLESLAAYAADLKGTMPISGRQEYLEAVMNDILFTEEK | |
|  |  |

| **Table S4**. Final optical density data of microculture growth of strain GGY018 expressing xylose isomerases, after 216 hours of cultivation. | | |  |
| --- | --- | --- | --- |
| **Sample** | **YNBX 5% xylose** | **YPX 5% xylose** | |
| **Empty vector (EV)** | 0.14 ± 0.06^E^ | 0.30 ± 0.01^E^ | |
| ***Orpinomyces sp. ukk1 (OrpXI)*** | 0.69 ± 0.04^A^ | 0.96 ± 0.02^A^ | |
| **XI09 Putative *Bacilli bacterium*** | 0.29 ± 0.01^E^ | 0.52 ± 0.02^D^ | |
| **XI10 Putative *Acholeplasmatales bacterium*** | 0.18 ± 0.01^E^ | 0.35 ± 0.09^E^ | |
| **XI11 Putative *Bacilli bacterium*** | 0.42 ± 0.02^C^ | 0.69 ± 0.03^C^ | |
| **XI12 Putative *Bacilli bacterium*** | 0.52 ± 0.06^B^ | 0.83 ± 0.01^B^ | |
| **XI13 Putative *Acholeplasmatales bacterium*** | 0.39 ± 0.01^C^ | 0.55 ± 0.01^D^ | |
| **XI14 Putative *Lachnospiraceae bacterium*** | 0.17 ± 0.01^D^ | 0.34 ± 0.01^E^ | |
| **XI15 Putative *Clostridia bacterium*** | 0.43 ± 0.02^BC^ | 0.59 ± 0.01^D^ | |
| Different superscript letters indicate significantly distinct groups (p<0.05), according to analysis of variance (ANOVA), for each parameter evaluated in the same treatment. | | |  |

| **Table S5**. Final optical density data of microculture growth of strain BVY271 expressing xylose isomerases, after 216 hours. | | | | |
| --- | --- | --- | --- | --- |
| **Sample** | **YNB 2% glucose** | **YNBX 5% xylose** | **YPD 2% glucose** | **YPX 5% xylose** |
| **Empty vector (EV)** | 0.72 ± 0.04^A^ | 0.10 ± 0.03^D^ | 1.03 ± 0.03^C^ | 0.17 ± 0.02^C^ |
| ***Orpinomyces sp. ukk1* (OrpXI)** | 0.76 ± 0.03^A^ | 1.05 ± 0.14^A^ | 1.05 ± 0.06^C^ | 1.29 ± 0.02^A^ |
| **XI09 Putative *Bacilli bacterium*** | 0.70 ± 0.06^A^ | 0.31 ± 0.03^C^ | 1.25 ± 0.05^A^ | 0.78 ± 0.28^B^ |
| **XI11 Putative *Bacilli bacterium*** | 0.74 ± 0.07^A^ | 0.88 ± 0.03^B^ | 1.25 ± 0.10^A^ | 1.29 ± 0.01^A^ |
| **XI12 Putative *Bacilli bacterium*** | 0.73 ± 0.02^A^ | 0.77 ± 0.01^B^ | 1.23 ± 0.09^AB^ | 1.24 ± 0.03^A^ |
| **XI13 Putative *Acholeplasmatales bacterium*** | 0.68 ± 0.05^A^ | 0.29 ± 0.02^C^ | 1.16 ± 0.05^AC^ | 1.11 ± 0.08^A^ |
| **XI15 Putative *Clostridia bacterium*** | 0.71 ± 0.03^A^ | 0.28 ± 0.02^C^ | 1.07 ± 0.05^BC^ | 0.75 ± 0.24^B^ |
| Different superscript letters indicate significantly distinct groups (p<0.05), according to analysis of variance (ANOVA), for each parameter evaluated in the same treatment. | | | | |

| **Table S6**. Final optical density data of microculture growth of strain BVY271 expressing novel xylose isomerases in minimal medium supplemented with Fe^2^, Mg^2^ and Mn^2^, after 216 hours. | | | | |
| --- | --- | --- | --- | --- |
| **Sample** | **YNBX** | **YNBX + Fe^2+^** | **YNBX + Mg^2+^** | **YNBX + Mn^2+^** |
| **Empty vector (EV)** | 0.10 ± 0.03^A^ | 0.08 ± 0.02^A^ | 0.08 ± 0.03^A^ | 0.06 ± 0.03^A^ |
| ***Orpinomyces sp. ukk1* (OrpXI)** | 1.05 ± 0.14^AB^ | 1.19 ± 0.01^A^ | 1.00 ± 0.01^B^ | 0.99 ± 0.04^B^ |
| **XI09 Putative *Bacilli bacterium*** | 0.31 ± 0.03^A^ | 0.33 ± 0.05^A^ | 0.32 ± 0.03^A^ | 0.30 ± 0.02^A^ |
| **XI11 Putative *Bacilli bacterium*** | 0.88 ± 0.03^B^ | 1.14 ± 0.01^A^ | 0.83 ± 0.02^C^ | 0.88 ± 0.02^BC^ |
| **XI12 Putative *Bacilli bacterium*** | 0.77 ± 0.01^B^ | 1.09 ± 0.01^A^ | 0.73 ± 0.02^BC^ | 0.68 ± 0.04^C^ |
| **XI13 Putative *Acholeplasmatales bacterium*** | 0.29 ± 0.02^A^ | 0.28 ± 0.02^A^ | 0.28 ± 0.02^A^ | 0.27 ± 0.02^A^ |
| **XI15 Putative *Clostridia bacterium*** | 0.28 ± 0.02^A^ | 0.29 ± 0.03^A^ | 0.26 ± 0.02^A^ | 0.31 ± 0.05^A^ |
| The analysis of the treatments was performed individually for each strain. Different superscript letters indicate significantly distinct groups (p<0.05) for each parameter evaluated. | | | | |
